# Binding of guide piRNA triggers methylation of the unstructured N-terminal region of Aub leading to assembly of the piRNA amplification complex

**DOI:** 10.1038/s41467-021-24351-x

**Published:** 2021-07-01

**Authors:** Xiawei Huang, Hongmiao Hu, Alexandre Webster, Fan Zou, Jiamu Du, Dinshaw J. Patel, Ravi Sachidanandam, Katalin Fejes Toth, Alexei A. Aravin, Sisi Li

**Affiliations:** 1 Division of Biology and Biological Engineering, Pasadena California Institute of Technology, Pasadena, CA USA; 2 National Key Laboratory of Plant Molecular Genetics and Shanghai Center for Plant Stress Biology, Center for Excellence in Molecular Plant Sciences, Chinese Academy of Sciences, Shanghai, China; 3 University of Chinese Academy of Sciences, Beijing, China; 4 Department of Biology, Southern University of Science and Technology of China, Shenzhen, China; 5 Structural Biology Program, Memorial Sloan Kettering Cancer Center, New York, NY USA; 6 Department of Oncological Sciences, Icahn School of Medicine at Mount Sinai, New York, NY USA; 7 Department of Biochemistry and Molecular Biology, International Cancer Center, Shenzhen University Health Science Center, Shenzhen, China; 8 Present Address: Department of Meiosis, Max Planck Institute for Biophysical Chemistry, Göttingen, Germany

**Keywords:** Piwi RNAs, X-ray crystallography

## Abstract

PIWI proteins use guide piRNAs to repress selfish genomic elements, protecting the genomic integrity of gametes and ensuring the fertility of animal species. Efficient transposon repression depends on amplification of piRNA guides in the ping-pong cycle, which in *Drosophila* entails tight cooperation between two PIWI proteins, Aub and Ago3. Here we show that post-translational modification, symmetric dimethylarginine (sDMA), of Aub is essential for piRNA biogenesis, transposon silencing and fertility. Methylation is triggered by loading of a piRNA guide into Aub, which exposes its unstructured N-terminal region to the PRMT5 methylosome complex. Thus, sDMA modification is a signal that Aub is loaded with piRNA guide. Amplification of piRNA in the ping-pong cycle requires assembly of a tertiary complex scaffolded by Krimper, which simultaneously binds the N-terminal regions of Aub and Ago3. To promote generation of new piRNA, Krimper uses its two Tudor domains to bind Aub and Ago3 in opposite modification and piRNA-loading states. Our results reveal that post-translational modifications in unstructured regions of PIWI proteins and their binding by Tudor domains that are capable of discriminating between modification states is essential for piRNA biogenesis and silencing.

## Abstract (web summary)

PIWI protein contains arginine rich motifs that are post-translationally modified to symmetrically methylated arginine (sDMA) residues. Here the authors show that piRNA loading into Aub triggers sDMA modification which is recognized by Krimper to promote formation of Krimper-Aub-Ago3 complex for piRNA amplification in Drosophila.

## Introduction

The PIWI-interacting RNA (piRNA) pathway acts as a conserved defensive system that represses the proliferation of transposable elements (TEs) in the germline of sexually reproducing animals^1–7^. Loss of PIWI proteins causes derepression of transposons associated with gametogenesis failure and sterility in flies and mice^8–11^. PIWI proteins recognize transposon targets with help of the associated small (23–30 nt) non-coding RNA guides, piRNAs.

PIWI proteins belong to the conserved Argonaute protein family present in all domains of life^9,12,13^. Argonautes bind nucleic acid guides and share common domain architecture, all containing the conserved N, PAZ, MID, and PIWI domains^14–17^. The MID and PAZ domains bind the 5′ and 3′ ends of the guide RNA, respectively^15,18–22^. The PIWI domain contains an RNase-H-like fold with a conserved amino acid tetrad that endows Argonautes with endonuclease activity for precise cleavage of the target^15,23,24^. The degradation of complementary target mRNA by PIWI proteins can trigger the generation of new RNA guides in a process termed the ping-pong cycle. Ping-pong requires cooperativity between two PIWI molecules as the product resulting from target cleavage by one protein is passed to the other and is converted to a new piRNA guide. In *Drosophila*, two distinct cytoplasmic PIWI proteins, Aub and Ago3, cooperate in the ping-pong cycle with each protein generating an RNA guide that is loaded into its partner. Amplification of piRNA guides through the ping-pong cycle is believed to be essential for efficient transposon repression as it allows the pathway to mount an adaptive response to actively transcribed transposons^2,5,25^.

In addition to four conserved domains, eukaryotic members of the Argonaute family, including PIWI proteins, contain an N-terminal extension region of various lengths with low sequence conservation. Structural studies of Agos suggest that the N-terminal regions adopt a disordered conformation^17,26–29^. Despite low overall conservation, the N-terminal region of the majority of PIWI proteins harbors arginine-rich (A/G)R motifs. In both insects and mammals, these motifs were shown to be substrates for post-translational modification by the PRMT5 methyltransferase, which produces symmetrically dimethylated arginine (sDMA) residues^30–34^. Loss of *Prmt5* (encoded by the Csul and Vls genes) in *Drosophila* leads to reduced piRNA level and accumulation of transposon transcripts in germ cells, suggesting that sDMA modification of PIWIs plays an important role in the piRNA pathway.

Multiple Tudor domain-containing proteins (TDRDs) can bind to sDMA modifications. Aromatic residues in binding pocket of Tudor domains form cation-π interactions with sDMA^35–38^. Studies in *Drosophila* and mouse revealed that several TDRDs interact with PIWIs and are involved in piRNA-guided transposon repression, although their specific molecular functions remain poorly understood^34,38–41^. Previously we found that the Tudor-domain containing protein Krimper is required for ping-pong piRNA amplification and is capable of both self-interactions and binding of the two ping-pong partners, Aub and Ago3^42^. Krimper co-localizes with Aub and Ago3 in nuage, a membraneless perinuclear cytoplasmic compartment where piRNA-guided target degradation and ping-pong are proposed to take place. Ago3 requires Krimper for recruitment into this compartment, though Aub does not. These results led to the proposal that Krimper assembles a complex that brings Ago3 to Aub and coordinates ping-pong in nuage^42^. However, the architecture of the ping-pong piRNA processing (4 P) complex and the extent to which Krimper regulates ping-pong remained unresolved.

Both the ping-pong cycle and sDMA modification of PIWI proteins are conserved features of the piRNA pathway, found in many organisms, suggesting that these processes are essential for pathway functions^2,5,31,39,43^. sDMA modification of PIWIs provides a binding platform for interactions with Tudor-domain proteins, however, its biological function and regulation are not known. Despite the essential role of ping-pong in transposon repression, we similarly have little understanding of the molecular mechanisms that control this process. Here we revealed the biological function of Aub and Ago3 sDMA modifications and show that it plays an essential role in orchestrating assembly of the 4 P complex in the ping-pong cycle. The modification signals whether PIWI proteins are loaded with guide piRNA, and this information is used to assemble a ping-pong complex that is receptive for directional transfer of RNA to an unloaded PIWI protein.

## Results

### Krimper can simultaneously bind Aub and Ago3 with specificity for methylation state using two separate Tudor domains

**Fig. 1:**
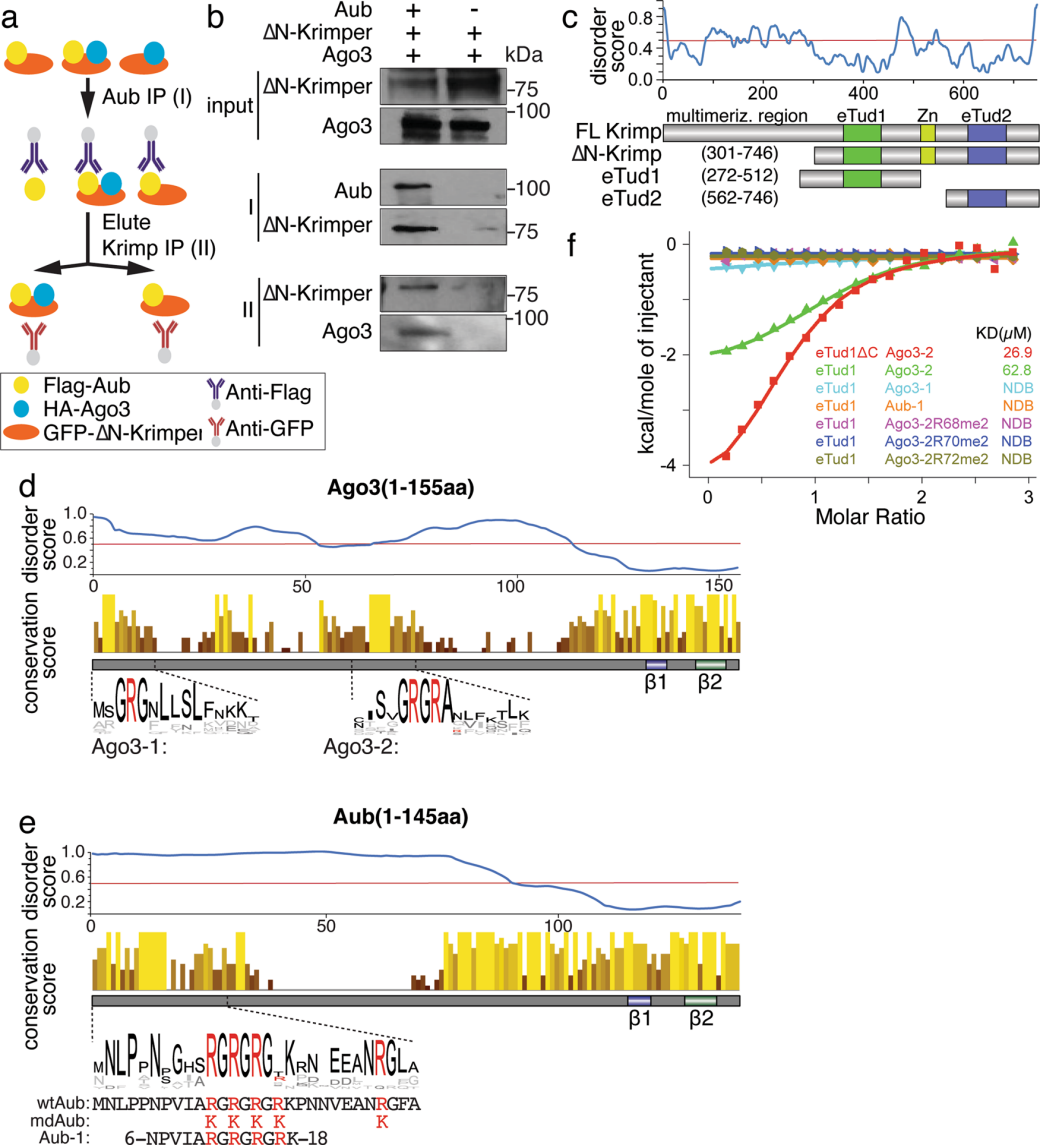
Krimper monomer simultaneously binds N-terminal regions of Aub and Ago3. **a** Scheme depicting two-step sequential immunoprecipitation. FLAG-tagged Aub, HA-tagged Ago3, and GFP-tagged truncated Krimper lacking its N terminal dimerization domain were co-expressed in the S2 cell. FLAG-immunoprecipitation was followed by elution and GFP IP. **b** Krimper monomer interacts with Aub and Ago3 simultaneously. Western blot detection of FLAG-Aub, HA-Ago3, and GFP-deltaN-Krimper in input and after the first FLAG- and second GFP-IP. Experiment reproduced twice with similar results. **c** Top: protein disorder prediction of Krimper. Bottom panel: domain architecture of Krimper and constructs used for ITC and structural analyses. **d** Conservation analysis of Ago3 (1-155aa). Top: disorder prediction of Ago3 (1-155aa). Middle: conservation score of each amino acid within Ago3(1-155aa). Conserved RG-repeat regions are. Bottom: peptides used for ITC and structural analysis. **e** Conservation analysis of Aub (1-145aa). Top: Disorder prediction of Aub (1-145aa). Middle: conservation score of each amino acid within Aub(1-145aa). The conserved RG-repeat region is enlarged. Bottom: sequence difference between wtAub and mdAub. Five arginines within the N terminus were replaced by lysines. Aub-1 peptide used for ITC and structural analysis. **f** eTud1 preferentially binds unmethylated Ago3. ITC analysis of Krimper eTud1 with different Ago3 or Aub peptides.

The essential step in the ping-pong cycle is the transfer of RNA between two PIWI proteins, Aub and Ago3. This process requires that the two proteins are in physical proximity to each other. Previously we found that Krimper binds both Aub and Ago3 suggesting that it might bring them into physical proximity to allow transfer of the RNA during ping-pong^42^. This might be possible through self-interaction of two Krimper molecules, each binding one PIWI protein, or by a single Krimper molecule simultaneously interacting with both Aub and Ago3. To discriminate between these two models, we employed two-step sequential immunoprecipitation to examine complex formation between Aub, Ago3, and a Krimper that lacks the N-terminal self-interaction region (residues 1-300aa) (Fig. 1a). We immuno-precipitated tagged Aub followed by elution of purified complexes and another round of immunoprecipitation against the self-interaction-deficient Krimper. If a single Krimper molecule is capable of simultaneously interacting with Aub and Ago3, then the final immunoprecipitated complexes should contain Ago3 in addition to Krimper and Aub. All three proteins were detected by Western blot after sequential purification (Fig. 1b). This indicates that a single Krimper molecule can concomitantly interact with both Aub and Ago3.

To understand how Krimper is able to bind Aub and Ago3 simultaneously, we analyzed its domain architecture. In addition to its self-interacting N-terminal region, Krimper contains two extended Tudor domains: eTud1(272-512) and eTud2 (562-746) (Fig. 1c). Tudor domains were previously implicated in protein-protein interactions through specific binding of methylated Arginine or Lysine residues^35–37^. PIWI proteins, including Aub and Ago3, harbor conserved symmetrically dimethylated Arginine (sDMA) residues embedded into RA/RG-motifs in their N-terminal extended regions^32,34,44^ In agreement with published structures of other PIWI and Ago proteins, which suggest that N-terminal extensions of these proteins adopt an unstructured conformation^16,17,26,27,45,46^, the N-terminal extended regions of both Aub and Ago3 have high levels of predicted disorder (Fig. 1d, e). Remarkably, RA/RG motifs, which are subject to sDMA modification are the only conserved sequences in the N-terminal regions of Aub and Ago3. The N-terminal regions are otherwise highly variable even between closely related *Drosophila* species (Fig. 1d, e). We previously found that the Krimper/Aub interaction depends on Aub methylation and found that the eTud2 domain of Krimper specifically binds sDMA-modified Aub^42^. However, in contrast to Aub, Ago3 was shown to interact with Krimper in its unmethylated state^42,47^, raising the question of how these two proteins interact.

Previous studies revealed that the N-terminal (1-83aa) sequence of Ago3 is both necessary and sufficient for Krimper binding^47^. However, the region of Krimper responsible for Ago3 binding remained unknown^42^. To study the Ago3-Krimper interaction, we expressed and purified eTud1 and eTud2 domains of Krimper (Fig. 1c) and tested their binding to peptides derived from the N-terminal Ago3 sequence using isothermal titration calorimetry (ITC) (Fig. 1f and Supplementary Fig. 1a). We found that eTud1 binds to the Ago3-2 peptide (aa residues 63-78 of Ago3, which includes the conserved RA/AG motif) with a binding affinity of 62.8 μM (Fig. 1f). Importantly, eTud1 does not detectably interact with the Aub-1 peptide (Fig. 1f). Thus, the two Tudor domains of Krimper have different binding preferences ensuring that Aub and Ago3 can bind Krimper simultaneously without direct competition for a binding site.

The Aub-Krimper (eTud2) interaction requires methylation of at least one of five Arg residues positioned within the RA/RG motif of the Aub N-terminal region^42^. Remarkably, the Ago3-2 peptide that binds the eTud1 domain also contains three Arg residues (R68, 70, and 72) within the RG/RA motif (Fig. 1d), which can be methylated in vivo, however, methylation is not required for Ago3 binding to Krimper (eTud1) (Fig. 1f). In fact, in agreement with in vivo experiments that indicate that Ago3 is bound to Krimper in its unmethylated state^42,47^, methylation of any of the three Arg residues disrupts binding of the Ago3-2 peptide to eTud1 (Fig. 1f).

Our results indicate that sDMA-modified Aub interacts with eTud2, while unmodified Ago3 interacts with eTud1, however, we previously reported that Aub co-immunoprecipitates with both eTud2 and N + eTud1 fragments^42^. To investigate this apparent discrepancy, we have repeated co-IP experiments and tested additional Krimp fragments. The new results confirmed our previous observation that Aub coimmunoprecipitates with the N + eTud1 fragment, however, we also found that the eTud1 fragment without N-terminal domain does not co-IP with Aub (Supplementary Fig. 1b). These results demonstrate that eTud1 does not bind Aub, confirming its preference toward Ago3, while they also show that the presence of the self-interacting N-terminal region is indispensable for co-immunoprecipitation of Aub with the N + eTud1 fragment. The most parsimonious explanation for these results is that formation of the complex between Aub and N + eTud1 observed in co-immunoprecipitation experiments is mediated by the full-length Krimper protein expressed in S2 cells that binds the N + eTud1 fragment through its self-interacting N-region.

Overall, our results indicate that binding of both Aub and Ago3 to Krimper is mediated by the RG/RA-motifs embedded in their unstructured N-terminal extended regions. However, the two proteins interact with Krimper in opposite modification states: Aub must be sDMA-modified, while Ago3 remains unmethylated. The distinct specificities of the two Tudor domains explain how Krimper can simultaneously bind Aub and Ago3 to bring them into physical proximity for ping-pong.

### Structural differences in the two Tudor domains of Krimper explain their different binding preference towards Arg-modified Aub and unmodified Ago3

**Fig. 2:**
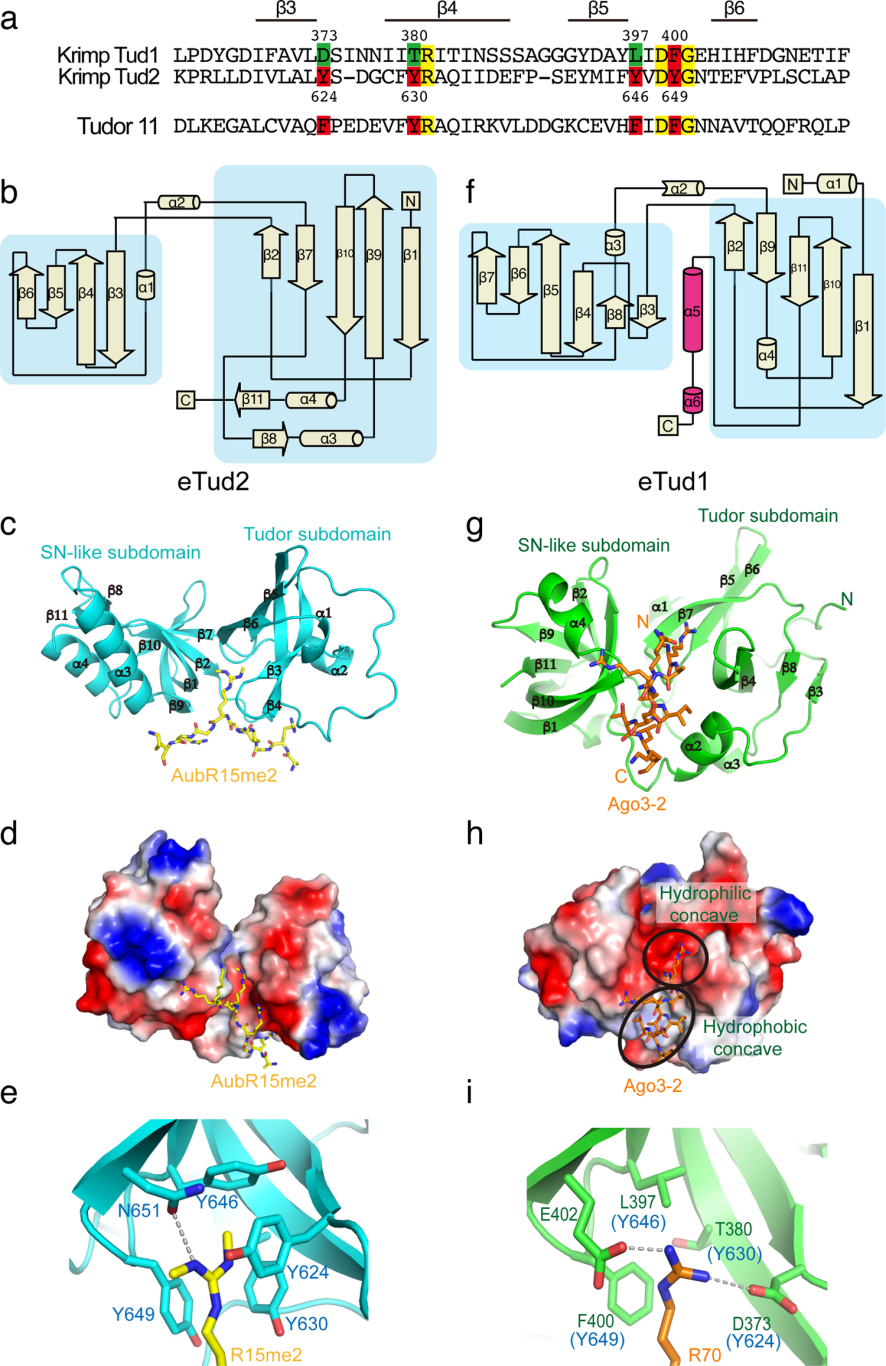
Structure of Krimper eTud2-Aub and eTud1-Ago3 complex. **a** Krimper contains the canonical eTud2 domain and the non-canonical eTud1 domain. Alignments of Krimper Tudor domains with Tudor1 domain 11 are shown. Residues forming the me-Arg binding pocket (red) and corresponding residues in tud1 (green) are shown. Additional conserved residues marked yellow. Structural features are shown above. **b** Overall topology demonstration of Krimper eTud2 domain. The Tudor and SN-like subdomain are indicated. **c** Overall structure of the Krimper eTud2-Aub-R15me2 complex with the bound Aub-R15me2 peptide shown in yellow. **d** Electrostatic surface of eTud2 with bound Aub-R15me2 peptide shown in yellow. **e** Enlarged view of the eTud2 aromatic cage with R15me2 shown in yellow. **f** Overall topology demonstration of Krimper eTud1 domain. The Tudor and SN-like subdomain are indicated. The latch helix is marked magenta. **g** The overall structure of the Krimper eTud1-Ago3-2 complex with bound Ago3-2 peptide shown in orange. **h** Electrostatic surface of eTud1 with bound Ago3-2 peptide shown in orange. **i** Enlarged the view of the eTud1 binding pocket with inserted Ago3 R70 residue indicated in orange.

To gain a detailed understanding of the interaction between Krimper (eTud2) and Aub, we undertook structural studies. We solved the crystal structure of the Krimper (eTud2) domain in complex with the Aub-R15me2 peptide that showed the highest binding affinity in ITC experiments^42^. In the 2.7 Å structure the eTud2 domain adopted a typical Tudor subdomain and a staphylococcal nuclease (SN)-like subdomain (Tudor-SN) architecture (Fig. 2a–c). sDMA-modified Arg-containing peptide binds within a negatively-charged cleft between the Tudor and SN domains, and is specifically recognized by a conserved aromatic cage consisting of four aromatic amino acids Tyr624, Tyr630, Tyr646, and Tyr649 (Fig. 2a–e), stabilized further by hydrophobic and cation-π interactions (Fig. 2e). In addition, eTud2 Asn651, which is conserved amongst other extended Tudor domains, forms a hydrogen bond with the guanidyl nitrogen of the methylated Arg15 (Fig. 2e and Supplementary Fig. 2a). Besides Arg15, other flanking arginine residues in the (G/A)R motif of Aub also contribute to protein-peptide binding (Supplementary Fig. 2a). Together, these structural features of eTud2 explain its specific binding to sDMA-modified Aub.

Unlike eTud2, Krimper (eTud1) has an unusual binding specificity towards the unmethylated Ago3 sequence containing the (G/A)R motif. To elucidate the interaction between eTud1 and Ago3 we solved the crystal structure of eTud1 in both apo and peptide bound forms. In the 2.1 Å eTud1 apo structure, crystals belong to the *P*2_1_2_1_2_1_ space group with two monomers in the asymmetric unit (Supplementary Table 1). The overall structure is similar to eTud2 apart from the C-terminal region (484-511aa) that forms a long α-helical fold that inserts into the narrow interface between the Tudor and SN-like subdomains. We named this novel C-terminal helical topology the “latch helix” (Fig. 2f and Supplementary Fig. 2b). The latch helix is stabilized through formation of extensive hydrogen bondings and salt bridge interactions with its amphipathic concave binding site within eTud1. Detailed interaction patterns and paradigms are shown in Supplementary Fig. 2c.

Next, we determined the crystal structure of the eTud1-Ago3-2 peptide complex at 2.4 Å resolution (Supplementary Table 1). The structure of eTud1 in the eTud1-Ago3-2 peptide complex exhibits almost an identical fold to the apo form, with an overall root mean square deviation of 0.60 Å (Supplementary Fig. 2d, e). However, the Ago3 peptide displaces the “latch helix” and adopts an α-helical structure to bind in the same cleft between the Tudor and SN-like subdomains, but with reverse directionality compared to the “latch helix” (Fig. 2g and Supplementary Fig. 2d, e). These results suggest that the “latch helix” might regulate the binding of Ago3 to eTud1. Indeed, ITC results showed that eTud1 devoid of the latch helix (named eTud1 ΔC) has a higher binding affinity towards the unmethylated Ago3-2 peptide (Fig. 1f).

The Ago3 peptide binds both a hydrophilic concave and a hydrophobic concave region in the cleft between the Tudor and SN-like subdomains of eTud1 (Fig. 2h). However, instead of a conserved aromatic cage that interacts with sDMA in other canonical Tudor domains including eTud2, the binding of unmethylated Arg70 of Ago3 in eTud1 is provided by a mostly hydrophilic pocket composed of three non-aromatic amino acids (Asp373, Thr380, and Leu397) (Figs. 2h, i). Arg70 forms hydrogen bonds with Asp373 and Glu402 in eTud1, supplemented by a cation-π interaction with Phe400 (Fig. 2i). Many extended Tudor domains adopt an incomplete aromatic cage, which may prefer unmethylated arginine peptides. In human TDRD2 protein, replacement of one of the four aromatic amino acids in the aromatic cage by a leucine residue, results in the preferential binding of unmethylated (A/G)R repeat with higher affinity^48^. In addition, the backbone of Arg68 and the side chain of Arg72 form hydrogen bonds with Asn376 and Thr445, respectively (Supplementary Fig. 2f). Notably, the side chain of Phe400 is rotated by 90° to form a stronger cation-π interaction with Arg70 when compared with the structure in the apostate (Supplementary Fig. 2g). Adjacent to the hydrophilic concave region there exists a hydrophobic concave region formed by Phe400, Trp339, and Tyr446. The Ago3 peptide forms a hydrophobic contact with the hydrophobic concave region utilizing Leu73 and Leu77 that strengthens its interactions (Fig. 2h and Supplementary Fig. 2f). The bound unmethylated arginine peptide in the Krimper eTud1 complex adopts the same directionality and similar localization as its counterpart in the TDRD2 complex (PDB code: 6B57) (Fig. 2h and Supplementary Fig. 3a, b). However, the peptide-binding interface of eTud1 is much narrower compared with the extended and negatively charged interface of TDRD2. Thus, eTud1 specifically recognizes unmodified R(A/G) motif in Ago3 using a unique hydrophilic concave and a hydrophobic concave region in the interface between the Tudor and SN-like subdomains. On the other hand, the N-terminal of the Aub sequence with an additional RG motif appears to be more hydrophilic and may not readily fit into the narrow hydrophobic concave cleft of eTud1, while the peptide of Ago3-1 with only one RG motif appears to be unable to form strong interactions with eTud1.

Although the overall topology of eTud1 is similar to other extended Tudor domains, eTud1 has its own unique folding and recognition characteristics. First, the N-terminus of eTud1 contains a long-loop (spanning amino acids 272-300) connecting and interacting with both the Tudor and the SN-like subdomains (Supplementary Fig. 3c). Pro292 within this N-terminal loop forms a CH-π interaction with Tyr365, Glu297 in the loop region forms a hydrogen bonding with the backbone of Ser387, Arg300 in the loop forms a hydrogen bond and salt bridge interactions with Asp394, while Tyr304 in the loop forms cation-π interaction with Arg428 in the SN-like domain. All these intramolecular interactions help to stabilize the overall structure of eTud1 (Supplementary Fig. 3c). Second, the linker helix α2 is shorter and shifted towards the Tudor-SN interface compared with other extended Tudor domains (PDB code: 3OMC), facilitating the interactions with the C-terminal “latch helix” or bound Ago3-2 peptide (Supplementary Fig. 3d).

Overall, our results reveal that the two Tudor domains of Krimper have distinct architectures that are responsible for the differential binding of two PIWI proteins. Aub and Ago3 have a similar organization, with N-terminal (G/A)R motifs that are subject to sDMA modification in vivo. However, while Aub interacts with the eTud2 domain in a methylated state through sDMA binding to a conserved aromatic cage, Ago3 binds the eTud1 domain in an unmethylated state employing a binding pocket that is distinct from other Tudor domain interactions. Furthermore, eTud1 possesses a ‘latch helix’ that must move out of the cleft to allow Ago3 binding providing a potential regulatory mechanism. These results highlight the crucial role that sDMA modifications of PIWI proteins play in regulating the formation of the tertiary ping-pong complex.

### sDMA modification of Aub is required for piRNA biogenesis through the ping-pong cycle but is dispensable for loading of piRNA into Aub

**Fig. 3:**
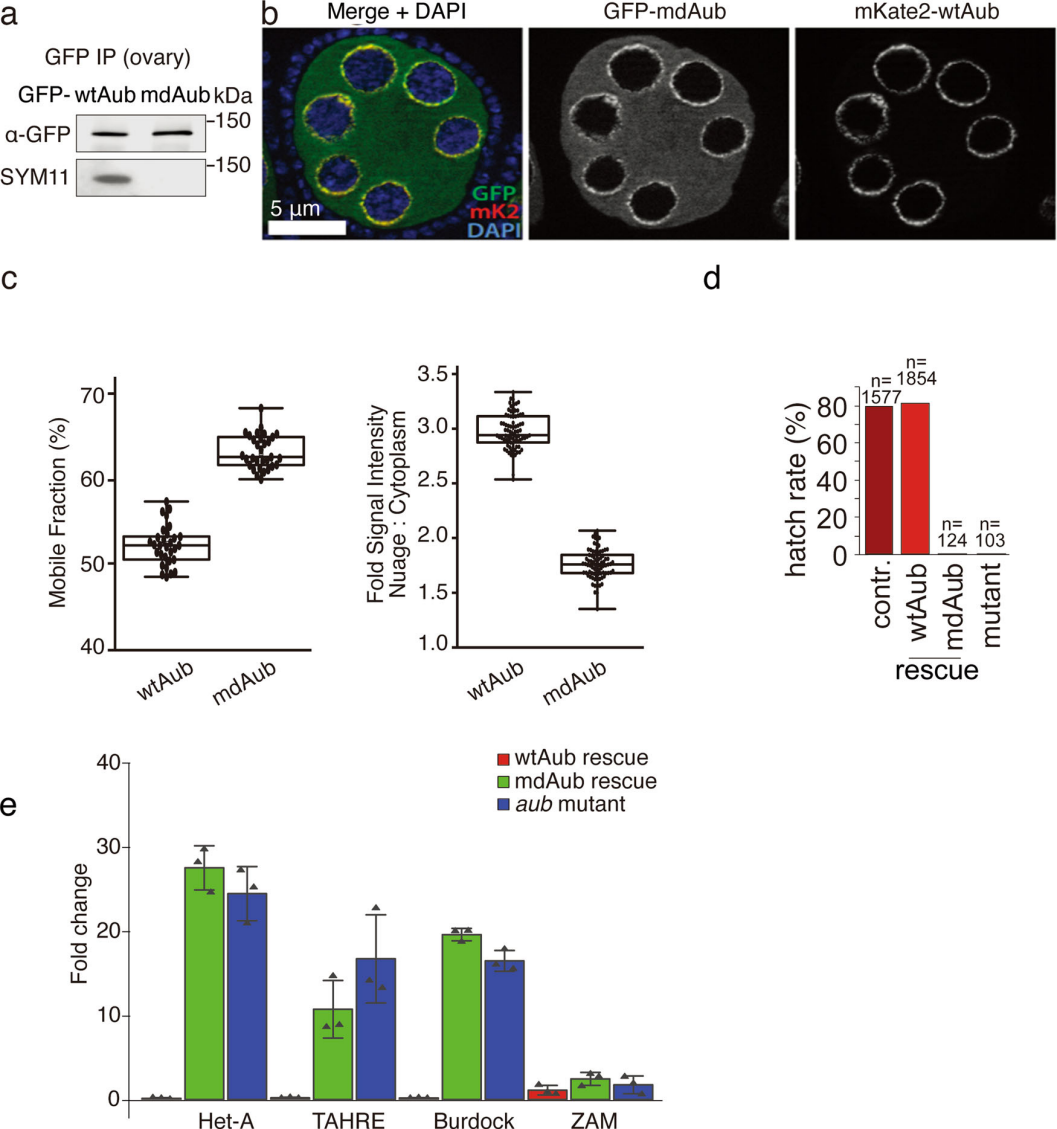
Arginine methylation of Aub is required for fertility and TE repression. **a** Mutating conserved N-terminal Arginines to Lysines (mdAub) leads to loss of symmetric dimethylation of Aub. A similar amount of wtAub and mdAub was immunoprecipitated from ovaries. Methylation was detected by Western blot using the SYM11 antibody. Experiment reproduced twice with similar results. **b** In the wildtype background, mdAub colocalizes with wildtype Aub in nuage. GFP-mdAub and mKate2-wtAub were co-expressed in ovaries under the control of the endogenous Aub promoter. Scale bar: 5 μm. Ten independent ovaries were used for imaging with similar results. **c** Arginine methylation deficiency slightly increases the fraction of mobile and dispersed cytoplasmic Aub protein. Left: the mobile fraction of nuage-localized GFP-wtAub and GFP-mdAub was determined in replicate FRAP experiments (constructs as in Extended Data Fig. 4c). (GFP-wtAub: *n* = 30 nurse cells. GFP-wtAub: *n* = 31 nurse cells). Experiments showed similar fractions of mobile proteins respectively. Right: microscopic quantification of the ratio of GFP signal originating from nuage versus cytoplasm is shown. (GFP-wtAub: *n* = 81 nurse cells. GFP-mdAub: *n* = 83 nurse cells). Experiments showed a similar ratio respectively. All transgenes are expressed under the control of the endogenous Aub promoter. Box plots present min to max, median, and all points. **d** Arginine methylation of Aub is required for fertility. The hatching rate of heterozygous control, Aub^HN/QC^ mutant females, and mutant females rescued with wt- or mdAub transgenes are indicated as % of eggs laid. **e** Arginine methylation of Aub is required for TE suppression. Fold changes of different TE transcripts in ovaries of flies with the indicated genotypes compared to the heterozygous control as measured by RT-qPCR. *n* = 3 biologically independent experiments with similar results. Data are presented as mean values ± sd.

Alongside previous studies, our results demonstrate the critical function that the methylation states of Aub and Ago3 play in their binding to Krimper. In order to investigate the function of sDMA modification of Aub in the broader piRNA pathway in vivo, we generated transgenic flies expressing a methylation-deficient version of Aub (mdAub) by mutating five Arg into Lys in the N-terminal region (Fig. 1e). The methylation of wild-type Aub was readily detected using Western blotting with SYM11 antibody that recognizes sDMA residues (Fig. 3a). In contrast, mdAub produced no signal, indicating that it indeed lacks sDMA modifications. Transgenic mdAub and control wild-type protein was expressed in the ovary at a similar level (Supplementary Fig. 4a). Wild-type Aub localized to nuage, the membrane-less subcellular compartment that surrounds the nucleus of nurse cells and is believed to play an essential role in piRNA biogenesis and TE repression. mdAub is also recruited to nuage, although compared to the wild-type protein a higher fraction is dispersed in the cytoplasm (Fig. 3b, c and Supplementary Fig. 4b). FRAP experiments show a slightly increased mobility of mdAub in nuage compared to wild-type Aub (Fig. 3c and Supplementary Fig. 4c). Thus, the methylation status of Aub does not affect protein stability and has only a minor effect on subcellular protein localization.

To explore the function of sDMA modification of Aub in vivo, we studied the ability of mdAub to rescue defects observed in Aub mutant flies. We expressed transgenic wild-type and methylation-deficient versions of Aub under the control of the endogenous Aub promoter and used genetic crosses to introduce these transgenes into the aub^HN/QC^ mutant background. Aub^HN/QC^ females are completely sterile due to severe defects in oocyte axis specification and DNA damage, presumably caused by derepression of multiple transposon families^49,50^. While the wild-type Aub protein was able to rescue transposon derepression and sterility of the *aub* mutant background, methylation-deficient Aub failed to rescue both phenotypes (Fig. 3d, e). The inability of mdAub to rescue sterility and transposon activation suggests that sDMA modification is essential for Aub function in vivo.

**Fig. 4:**
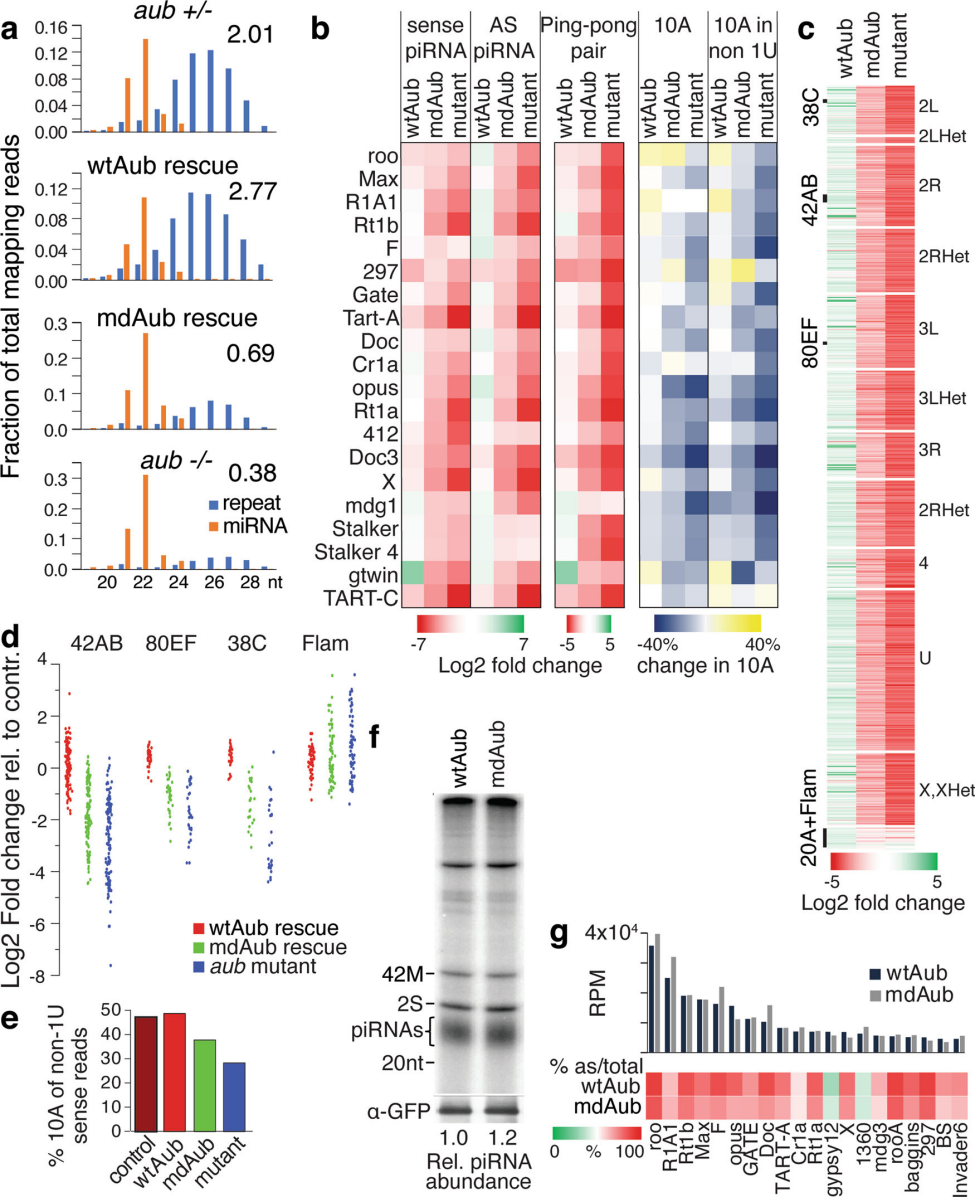
Arginine methylation of Aub is dispensable for piRNA binding but required for piRNA expression and ping-pong processing. **a** Length distributions of reads annotated as miRNAs and repeats in ovarian small RNA libraries from indicated flies. The ratio of repeat-derived piRNAs to miRNAs in each genotype is indicated above the graphs. **b** Arginine methylation of Aub is required for piRNA expression and ping-pong processing of transposon piRNA. The heatmap shows the fold change compared to control (aub heterozygotes) for the top 20 most abundant TE families present in the control ovary. The last two heatmaps show changes in 10 A bias for all sense piRNAs and non-1U sense piRNAs, respectively. **c** Aub sDMA modification is required for the generation of piRNAs from piRNA clusters. wtAub, but not mdAub rescues piRNA expression from genomic regions affected by aub mutation. Genomic windows with more than 5 RPM in control and more than 80% reduction in aub mutant are shown; uni-strand clusters flam and 20 A that are not affected in aub mutants are shown for comparison. **d** Detailed analysis of piRNA generation from major piRNA clusters. Reads uniquely mapping to four clusters were determined and plotted as the fold change compared to the het control. The dot represents 1 kb windows with uniquely mapping piRNAs within the clusters. **e** The fraction of piRNAs that have the signature of the ping-pong processing (piRNAs that map to TEs in sense orientation and have 10 A, but not 1U bias) is reduced in aub mutants and only partially rescued by mdAub expression. The graphs show the fraction of sense non-1U piRNAs that have 10 A in libraries from indicated ovaries. **f** Aub Arginine methylation is not required for piRNA binding. GFP-tagged mdAub and wtAub were immunoprecipitated from ovaries where the transgene was expressed in the wild-type background. Relative piRNA abundance normalized to IP-ed protein is estimated based on band intensity. Experiment reproduced twice with similar results. **g** Arginine methylation of Aub does not greatly affect the TE and antisense fractions of Aub-bound piRNAs. The bar chart shows normalized read counts (RPM) mapped to the 20 TE families with post abundant piRNAs. Aub transgenes were expressed on the wild-type background. Heatmap shows the % of antisense reads mapping to each TE family.

Sequencing of total small RNA libraries from ovaries of *aub* mutant females that express wild-type or methylation-deficient variants of Aub revealed that piRNAs mapping to different transposon families and major piRNA clusters were severely reduced in the *aub*^*HN/QC*^ mutant (Fig. 4a, b, d). While expression of methylation-deficient Aub increased piRNA levels, this increase was smaller than that observed in flies rescued with the wild-type protein. In ovaries of control (*aub* heterozygotes) flies piRNAs are twice as abundant as miRNAs, while this ratio drops to only 0.38 (more than fivefold) in *aub* mutants. Expression of wild-type Aub completely rescues piRNA expression, while expression of methylation-deficient Aub increases the ratio to 0.69, which is higher than the mutant, but still far from levels in the control (Fig. 4a). The analysis of piRNAs targeting individual TE families showed that expression of wild-type protein rescues piRNA loss for all families, while expression of mdAub provides intermediate piRNA level between the mutant and the control (Fig. 4b). Both sense and antisense piRNAs to individual TE families are reduced in flies expressing mdAub. To comprehensively analyze the effect of sDMA of Aub on piRNA biogenesis, we analyzed piRNA generation throughout the genome in 5 kb windows. Almost all regions that generate piRNAs with notable exception of uni-strand piRNA clusters such as *flam* and *20* *A* show dramatic loss of piRNA in the aub^HN/QC^ mutant (Fig. 4c). Unlike the wild-type protein, expression of mdAub fails to rescue piRNA generation throughout the genome indicating that sDMA is crucial for piRNA biogenesis (Fig. 4b–d).

We previously proposed that binding of Aub and Ago3 to Krimper assembles a ping-pong piRNA processing (4 P) complex that is essential for the ping-pong cycle^42^. To explore the role of Aub methylation in ping-pong we analyzed piRNA profiles of *aub* mutant flies rescued with either wild-type or mdAub to find signatures of ping-pong processing: pairs of piRNAs whose 5′ ends are complementary across 10 nt. In the piRNA libraries isolated from ovaries of mdAub-rescued flies, piRNAs with ping-pong processing signatures were reduced for almost all major families of transposons compared to those from the wild type Aub rescue (Fig. 4b), indicating defects in the heterotypic ping-pong cycle. Another signature of ping-pong processing is the presence of sense piRNAs that lack U at the first position but have a bias for A in position 10 (10 A bias). In total, 10 A bias in sense piRNAs was dramatically reduced for individual TE families in aub mutants. While the expression of wtAub rescued the bias, mdAub expression led to the only partial rescue (Fig. 4b). For all repeat-derived piRNAs, 10 A bias among sense non-1U piRNA is 47% in heterozygous control and drops to 28% in *aub* mutants. Expression of wild-type Aub rescued the bias to 49%, while expression of mdAub led to an intermediate value of 38% (Fig. 4e). Mutation of Aub and its rescue by wtAub and mdAub do not change other nucleotide biases in piRNA (Supplementary Fig. 5a).

To explore if disruption of piRNA biogenesis might be caused by an inability of mdAub to be loaded with piRNAs, we expressed the mdAub transgene in wild-type flies that also express endogenous wild-type Aub. These flies have normal fertility, indicating that the expression of mdAub does not induce a dominant-negative effect. Next, we purified mdAub and control wild-type Aub protein and radiolabeled isolated RNAs associated with both proteins. Quantification of the signal showed that small RNA loading of wild type and mdAub is similar (Fig. 4f). Small RNAs associated with the two proteins have a similar length distribution (Supplementary Fig. 5b). To explore if small RNA loaded in mdAub might be different from sequences loaded into wild-type Aub protein we cloned and analyzed small RNAs residing in both complexes. Similar to wild-type Aub protein, the majority of sequences in mdAub are antisense to various transposons families (Fig. 4g). Overall, we did not find any abnormalities in the amount and composition of piRNA loaded into mdAub when it was expressed in the wild-type Aub background. Thus, the methylation status of Aub plays no direct role in piRNA loading.

Amplification of piRNAs in the ping-pong cycle is dependent on the slicer activity of Aub. To test if the mdAub mutation has an effect on its slicer activity, we have performed in vitro cleavage assays with proteins purified from S2 cells. When loaded with guide RNA both wild-type and mdAub cleave complementary RNA with similar efficiency and precision indicating that mdAub exhibits normal slicer activity (Supplementary Fig. 4d).

Taken together, our results indicate that sDMA modification of Aub is essential for piRNA biogenesis through piRNA amplification in the ping-pong cycle and hence the repression of transposons. However, mdAub has a normal slicer activity and sDMA modification is not required for loading of piRNA into mdAub if cells also express wild-type Aub that ensures functional piRNA biogenesis. This finding corroborates our earlier proposal that Aub methylation is required for the formation of the Krimper, Aub, and Ago3 complex, which in turn is required for Ago3 loading^42^. Overall, our results indicate that the sDMA modification of Aub has a crucial role in piRNA biogenesis and specifically in the ping-pong amplification cycle.

### piRNA loading induces Aub methylation

**Fig. 5:**
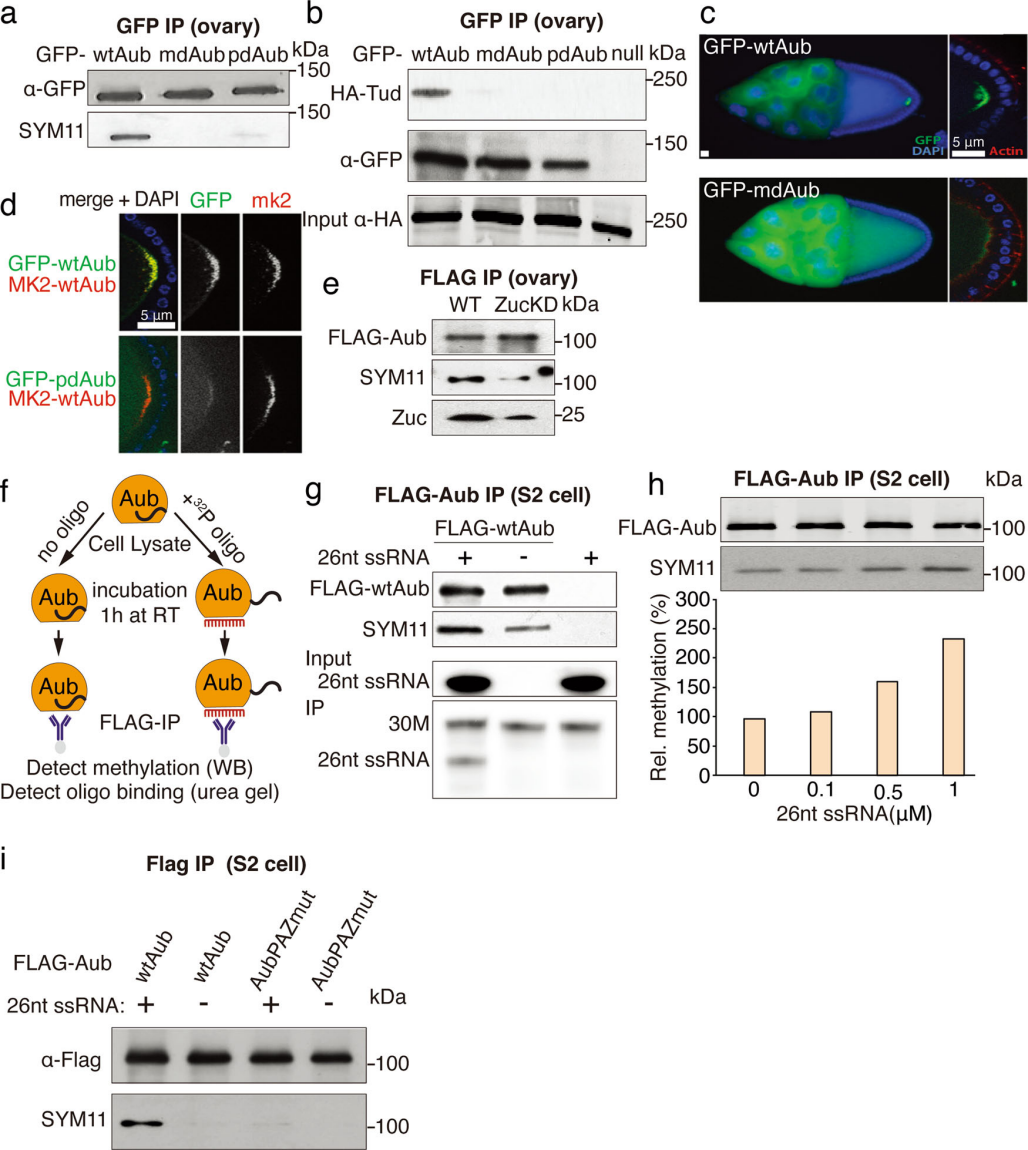
RNA binding promotes Aub arginine methylation. **a** RNA binding is required for Aub arginine methylation in vivo. Transgenes were expressed on the wildtype background. Total protein level and methylation was detected in Western blot. The experiment reproduced three times with similar results. **b** RNA binding is required for Aub interaction with Tudor in vivo. GFP tagged Aub was immunoprecipitated from ovarian lysate, followed by Western blot detection of the tagged transgenes. Experiment reproduced twice with similar results. **c** Arginine methylation is required for the localization of Aub to the pole plasm. GFP tagged wtAub and mdAub were separately expressed in ovaries on the wild-type Aub background. Scale bar: 5 μm. Ten independent ovaries were used for imaging with similar results. **d** RNA binding is required for Aub localization into the pole plasm. Upper panel: GFP-wtAub and mKate2-wtAub were co-expressed in ovaries in the wild-type Aub background. Bottom panel: GFP-pdAub and mKate2-wtAub were co-expressed in ovaries in the wild-type Aub background. Scale bar: 5 μm. Ten independent ovaries were used for imaging with similar results. **e** Zucchini KD leads to decreased Aub arginine methylation. FLAG-tagged wtAub was immunoprecipitated from control and Zuc KD ovaries. Methylation was detected by Western blot. Experiment reproduced twice with similar results. **f** Scheme of in vitro oligo-binding experiments. Lysate of S2 cells expressing FLAG-Aub was incubated with or without ^32^P labeled 26 nt RNA oligos followed by FLAG immunoprecipitation and detection of methylation by Western blot and oligo binding on urea gel. **g** RNA oligo binding promotes Aub sDMA modification. In total, 30 nt (30 M) ssRNA was spiked into each IP-ed sample to normalize for total IP-ed RNA amount. Experiment reproduced twice with similar results. **h** Aub methylation correlates with synthetic ssRNA concentration added to lysates of S2 cells expressing FLAG-Aub. Methylation was quantified based on band intensity in FLAG and SYM11 Western blot and normalized to the no oligo control. Experiment reproduced twice with similar results. **i** Binding of the RNA 3′ end by Aub’s PAZ domain is required for sDMA modification. Wild-type Aub and AubPAZmut were expressed in S2 cells. sDMA modification was detected by western blot using SYM11 antibody. Experiment reproduced twice with similar results.

Our results demonstrate that sDMA modification of Aub is essential for the ability of the piRNA pathway to generate a repertoire of guide piRNAs that effectively target and repress transposons. To understand the molecular mechanism of sDMA modifications we decided to explore if loading of piRNAs into Aub affects its modification. We used flies that express a piRNA-binding deficient version of Aub (pdAub) due to mutation in two conserved residues within the 5′ end binding pocket located in the MID domain^42^. We expressed pdAub on a wild-type background to preserve an intact piRNA biogenesis pathway and tested the methylation status of Aub using the SYM11 antibody that recognizes methylated Arginine residues. Remarkably, we found that methylation of pdAub is strongly reduced compared to the wild-type protein (Fig. 5a) suggesting that prior loading with piRNA is necessary for the post-translational sDMA modification of Aub. We employed a Tudor-Aub binding assay that strictly depends on the Aub methylation^44^ as another tool to assess the modification status of Aub. As expected, Tudor co-immunoprecipitated with wild-type Aub, but not mdAub, from ovary lysates (Fig. 5b). Similar to mdAub, pdAub did not bind Tudor protein confirming that Aub piRNA loading is a prerequisite for sDMA modification.

To further explore the link between piRNA loading and sDMA modification we tested Aub interaction with Tudor in situ, inside of the cells. In growing oocytes, Tudor protein localizes to the pole plasm, which is the posterior region of the oocyte essential for germ cell specification in the zygote^51–53^. Tudor binds sDMA-modified Aub leading to its accumulation in the pole plasm^54^. As expected, while wild-type Aub was concentrated in the pole plasm, mdAub failed to be properly localized because it could not interact with Tudor protein (Fig. 5c). Similarly, to methylation-deficient Aub, pdAub had strong defects in pole plasm localization (Fig. 5d) confirming that piRNA-free Aub is unable to be methylated.

The experiments described above suggest that a defect in piRNA loading impairs Aub methylation, however, it is possible that two point mutations introduced into pdAub to impair piRNA binding may cause additional structural changes that render it incapable of methylation. Therefore, we further explored the link between piRNA loading and sDMA modification using wild-type Aub protein. We analyzed the modification status of wild-type Aub in flies that have a low level of piRNAs due to knockdown of Zucchini (Zuc): a nuclease that is essential for piRNA processing^55–57^. Depletion of Zuc in fly ovaries by RNAi caused a reduction of Aub methylation (Fig. 5e), suggesting that the modification status of wild-type Aub depends on its piRNA loading status.

To directly test if binding of guide piRNA induces Aub modification, we studied if the loading of a chemically synthesized RNA in cell lysate triggers Aub methylation. Argonaute proteins (including members of the PIWI clade) can be loaded with 5′-monophosphorylated short RNA when incubated together in vitro^16,45,46^. We expressed tagged Aub in S2 cells that lack an active piRNA pathway and do not express piRNA. We added different amounts of 5′-monophosphorylated 26 nt RNA into the lysate of S2 cells that expressed Aub protein and incubated lysate for 1 h to allow Aub to be loaded with RNA and the methylation reaction to proceed. Next, we purified Aub and analyzed associated RNA and its modification status (Fig. 5f). The detection of bound RNA indicates that Aub binds exogenous short RNA during incubation (Fig. 5g). Importantly, the addition of increasing amounts of short RNA led to a progressive increase in Aub methylation (Fig. 5h). Thus, loading of Aub with synthetic RNA triggers sDMA modification in cell lysate, indicating a direct link between RNA binding and modification.

PIWI proteins bind mature piRNAs by anchoring both of its ends: the 5′ end is bound by the MID domain, while the 3′ end is anchored by the PAZ domain^58,59^. However, loading and final processing of piRNA guides are coupled, so that longer piRNA precursors are first anchored at their 5′ end, followed by Zuc mediated cleavage or trimming of their 3′ end before they can be anchored by the PAZ domain^56,57,60^. To explore if sDMA modification is triggered by 5′ end binding or requires complete binding of the guide at both ends, we generated another Aub mutant unable to bind the 3′ end, by mutating two conserved Tyr residues in its PAZ domain (Tyr345 and Tyr346) that were shown to be indispensable for this process^61^. Though this mutant has an intact 5′ end binding pocket, it has miniscule level of sDMA modification upon addition of the 26 nt synthetic RNA (Fig. 5i), indicating that complete binding of the guide—both 5′ and 3′ ends—is required to induce modification.

### piRNA loading induces conformational change and promotes Aub modification by increased accessibility of the N-terminal sDMA motif to the PRMT5 methylosome complex

How can piRNA loading into Aub induce its methylation? The binding of RNA might induce conformational changes in protein structure that expose previously hidden residues to the solvent and enhance access to the enzyme that catalyzes modification. Indeed, structural studies of Argonaute proteins demonstrated an extensive conformational change in protein structure upon binding to guide RNAs^16,62^. However, the structure of *Drosophila* Aub protein has yet to be solved and the N-terminal extension regions are not present in the available structures of PIWI proteins^45,46^. To study if the loading of RNA into Aub induces its conformation change, we employed the RNA loading strategy described in the previous experiment (Fig. 5f) followed by a partial proteolysis assay. Aub protein with a FLAG tag introduced at its N-terminus was expressed in S2 cells and half of the lysate was incubated with 1 µM of synthetic 26 nt RNA for 1 h, while RNA was omitted for the other half. After incubation, the protein was captured on anti-FLAG beads and subjected to serial dilution of chymotrypsin protease followed by SDS-PAGE gel electrophoresis (Supplementary Fig. 6a). Western blot with anti-FLAG antibody showed that the N-terminus of Aub has different accessibility to protease in free and RNA-bound forms (Supplementary Fig. 6a) suggesting that Aub undergoes a conformational change upon RNA loading.

**Fig. 6:**
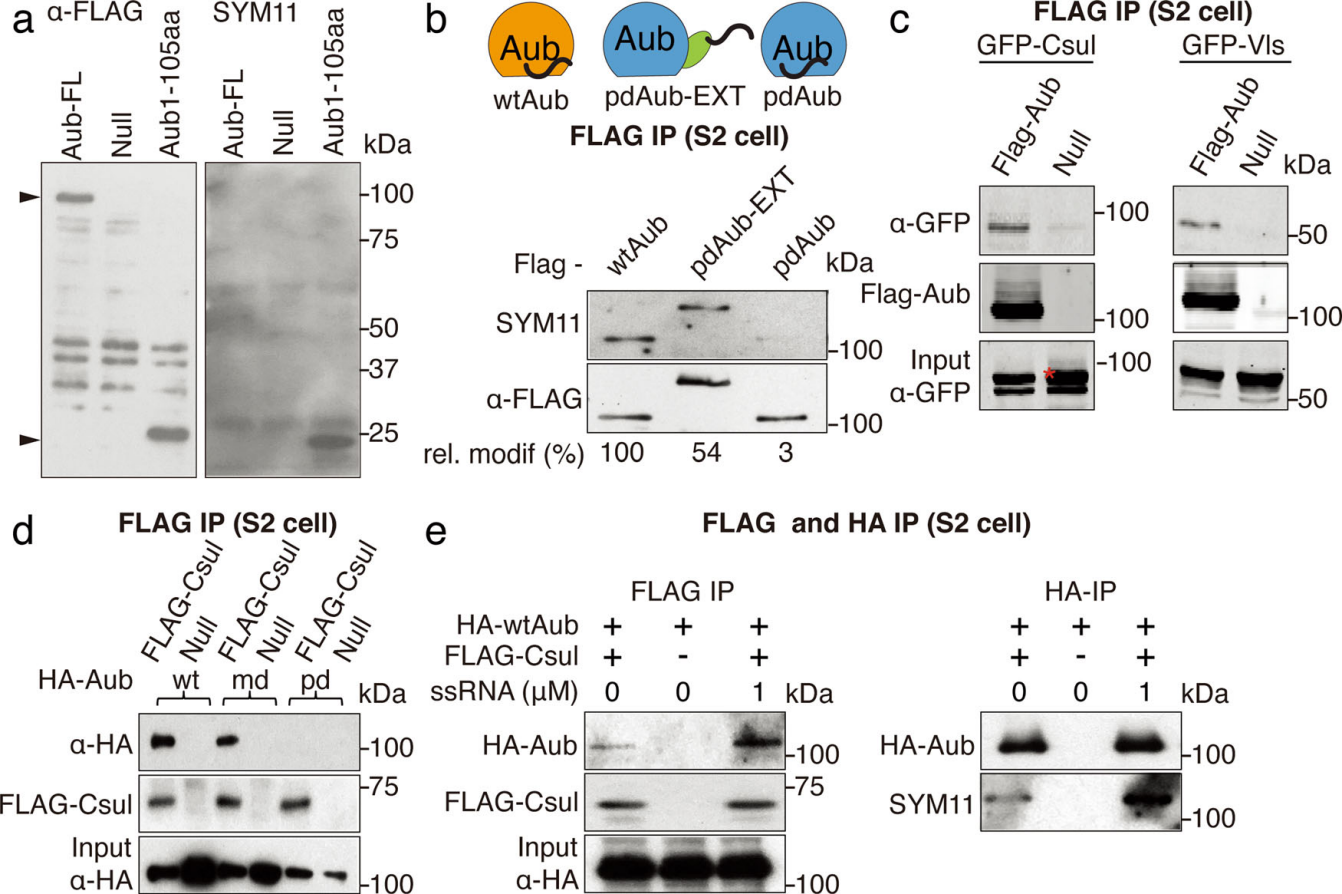
RNA binding triggers a conformational change in Aub, exposing its N terminus to the methyltransferase Csul/Vls complex. **a** N-terminal region within Aub protein is not easily accessible to methylation. FLAG-tagged full length and N-terminal truncated (1-105aa) Aub were expressed in S2 cells and immunoprecipitated. Total protein and methylation level were assessed by Western blot. Arrowheads indicate correct size for full-length and N-terminal fragment, respectively. Experiment reproduced twice with similar results. **b** Top: scheme showing architectures of different Aub constructs expressed and IP-ed from S2 cells. EGFP inserted between the N-terminal and PAZ domain (pdAub-EXT) artificially exposes N-terminus in absence of piRNA binding. Bottom: western blot analysis of methylation states. Relative methylation level as estimated by the ratio of SYM11/FLAG band intensities normalized to wildtype is listed. Experiment reproduced twice with similar results. **c** Aub interacts with Csul and Vls in S2 cells. Co-IP Western blot of tagged transgenes. Asterisk indicates band corresponding to GFP-Csul in the INPUT. **d** Aub binding to Csul depends on RNA binding but not on Arg methylation. FLAG-Csul and HA-Aub transgenes were expressed in S2 cells and coIP followed by Western detection. Experiment reproduced twice with similar results. **e** RNA loading of Aub leads to increased binding to Csul and increased methylation of Aub. FLAG-Csul and HA-Aub were expressed in S2 cells, lysates were incubated in the presence or absence of ssRNA oligo prior to coIP followed by Western detection. Experiment reproduced twice with similar results.

To further explore how conformation affects Aub methylation we compared the modification status of the full-length protein and N-terminal fragment that harbors the (G/A)R motif. Tagged full-length Aub and the N-terminus (1-105 aa) fragment were expressed in S2 cells and the methylation status was probed by Western blot with SYM11 antibody. Similar to previous experiments, only a very weak modification was observed on full-length protein, however, we detected strong methylation of the N-terminal fragment (Fig. 6a). This result suggests that the N-terminal region of Aub protein is not easily accessible to modification if the protein is not bound to guide RNA, but the same sequence is methylated strongly if it is apart from the whole protein.

Next, we tested if we can alter the accessibility of the N-terminal region to the methylation machinery by inserting an extra sequence between this region and the rest of the protein (named pdAub-EXT). While Aub deficient in piRNA binding was methylated only on a very weak level, insertion of a GFP sequence after AA105 caused robust methylation (Fig. 6b). Thus, insertion of an extended sequence between the N-terminus and the rest of the protein converts the N-terminal (GA)R motif into a good substrate for modification even if the protein is not loaded with RNA. Overall, these experiments suggest that the N-terminal region is not readily accessible if Aub is not bound to RNA, however, it can be readily methylated upon solvent exposure.

We tested the biochemical interactions between Aub and the components of the methylosome complex responsible for sDMA modifications. sDMA modifications are catalyzed by a conserved methylosome enzyme composed of *Drosophila* PRMT5 homolog Capsuleen (Csul) and MEP50 homolog Valois (Vls)^63–65^ and previous genetic studies implicated both proteins are required for Aub sDMA modifications^54,66,67^. We found that Csul and Vls co-purifies with Aub in S2 cells (Fig. 6c). We further dissected Aub protein into individual domains and found that the N-terminal fragment (1-220aa) alone was sufficient for binding to Csul, while deletion of this region disrupted the interaction (Supplementary Fig. 6b). This indicated that the methylosome interacts with the N-terminal region of Aub harboring (G/A)R motif, while other regions are dispensable for binding.

To study if piRNA loading affects the interaction of Aub with the methylosome complex we tested the binding of Csul with Aub mutants. Csul binds wild-type and mdAub indicating that arginine residues that are targeted for methylation are dispensable for interaction with Csul (Fig. 6d). However, Csul did not interact with the piRNA-deficient pdAub mutant (Fig. 6d) suggesting that conformation change induced by binding to RNA is required for interactions with Csul. Finally, we loaded wild-type Aub with synthesized 26 nt RNA and tested the effect of RNA binding on the interaction with Csul. Addition of synthetic RNA increased interaction of Aub with Csul and subsequent Aub methylation (Fig. 6e). Thus, we determined that increased levels of sDMA modifications upon RNA binding appear to be a direct result of the stronger binding of RNA-loaded Aub to the methylosome complex.

## Discussion

Ago proteins are present in all domains of life and use a conserved molecular architecture to bind guide nucleic acids and recognize and process complementary targets^14–17^. Despite the simplicity of the effector complex composed of one protein and one nucleic acid, Argonautes play crucial roles in the control of gene expression and have remarkably diverse sets of targets and functions. The diversity of post-translational modifications expands the regulation and function of Argonaute proteins^68–70^. We found that a post-translational modification specific to members of the PIWI clade of the Argonaute family, sDMA in the flexible N-terminal region, encodes information about guide RNA loading status and regulates interactions, cellular localization, and function of PIWI proteins.

Although PIWI proteins and piRNAs share many similarities with other Agos and their RNA guides, the piRNA pathway has evolved unique features that are essential for its function as an adaptive genome defense system. One such unique property is the amplification of piRNAs that target active transposons in the ping-pong cycle^2,5^. Ping-pong employs the intrinsic RNA binding and processing capabilities of Ago proteins, however, it creates new functionality through the cooperation between two PIWI proteins. Our results indicate that the ping-pong cycle and sDMA-modification are tightly linked and that the modification status of PIWI proteins regulates the assembly of the ping-pong processing complex.

### Molecular mechanism of sDMA regulation

Several lines of evidence suggest that sDMA modification of Aub is induced by the binding of a piRNA guide. First, Aub mutants that are deficient in piRNA binding due to mutation in either the RNA 5′ or the 3′ end binding pocket have a decreased level of sDMA modification (Fig. 5a, b, i). Second, disruption of piRNA biogenesis diminishes methylation of wild-type Aub (Fig. 5e). Finally, the loading of chemically synthesized RNA into Aub promotes its association with the methylosome complex (Fig. 6e) and sDMA modification (Figs. 5g–i and 6e). In contrast, sDMA modification of Aub is not required for its loading with piRNA (Fig. 4f, g and Supplementary Fig. 5b) and for its slicer activity (Supplementary Fig. 4d). Together, these results suggest that sDMA modification of Aub acts as a signal of its piRNA-bound state.

**Fig. 7:**
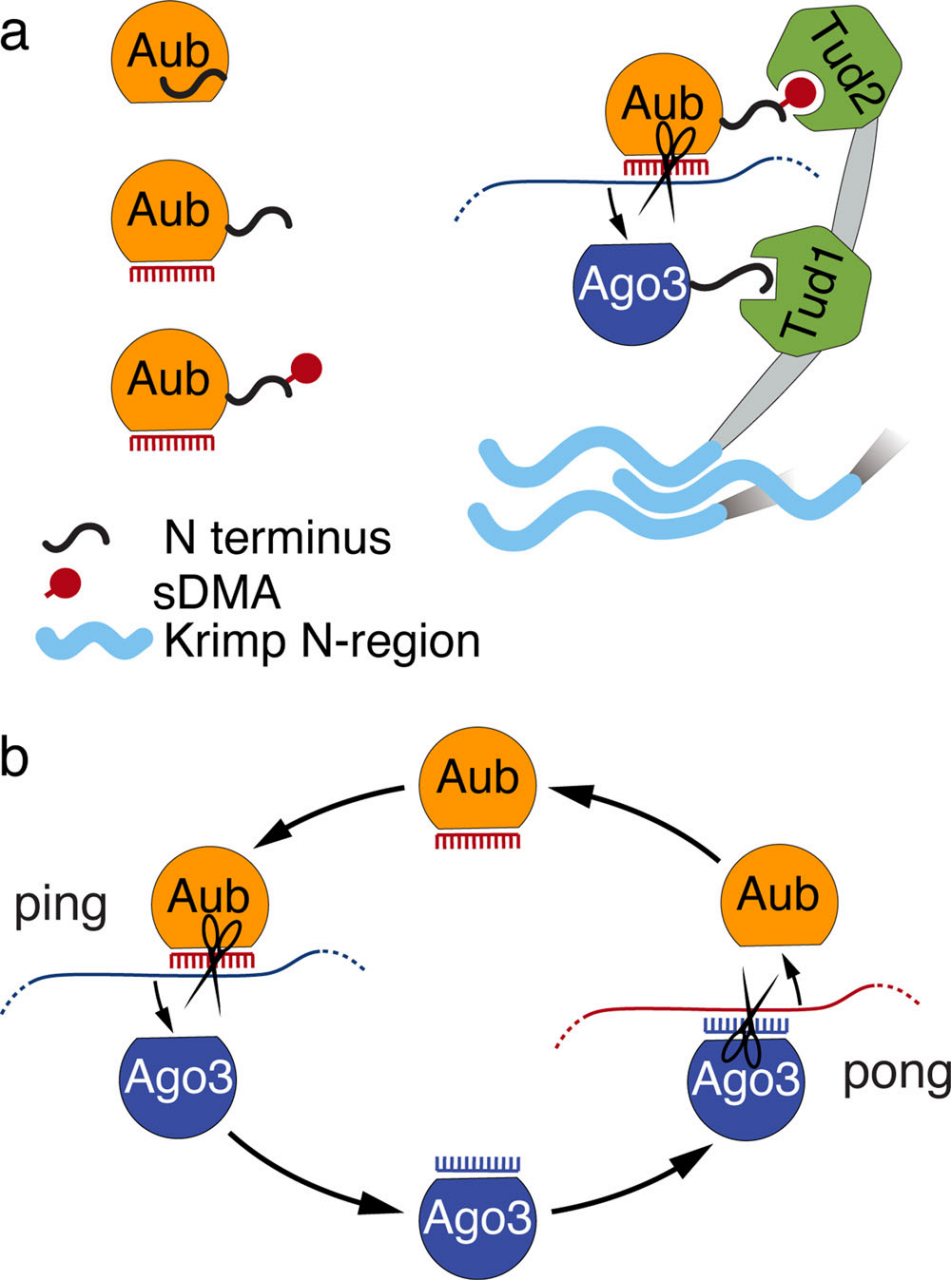
Model for sDMA regulation and its function in ping-pong cycle. **a** Model sDMA dependent assembly of the ping processing complex. The N-terminus of unloaded Aub is inaccessible. Binding to piRNA guide leads to a conformational change of Aub exposing its N terminus and enabling methylation of its N-terminal arginines. Methylation thus serves as a signal of loading-state and enables Aub binding to the Tud2 domain of Krimper, which specifically recognizes methylated Arginines. The Tud1 domain of Krimper binds unmethylated, unloaded Ago3, enabling Ago3 loading with the newly processed piRNA. The N-terminal unstructured region of Krimper allows Krimper multimerization resulting in a Krimper scaffold that might assist in nuage assembly and ensuring high local concentration of Aub and Ago3. **b** The Ping-Pong cycle consists of two distinct stages. In the ping stage, Aub with its piRNA guide targets piRNA precursors or TE transcripts. Cleaved, mature piRNA is loaded into Ago3. In the pong stage, piRNA-loaded Ago3 targets antisense piRNA precursors. Cleaved mature piRNA is loaded into Aub. The ping and pong processing could be accomplished by different complexes in nuage.

Our results suggest that piRNA loading induces sDMA methylation through a conformational change that makes the N-terminal sequence accessible to the methylation enzyme. While unloaded Aub is poorly methylated, the N-terminal sequence alone is a good substrate for methylation (Fig. 6a). Insertion of a sequence between the N-terminal region and the rest of the protein also promotes methylation (despite the protein not being able to bind piRNA), suggesting that other parts of the protein inhibit modification (Fig. 6b). Finally, partial proteolysis indicates that Aub undergoes a conformation change upon piRNA loading (Supplementary Fig. 6a). Combined, these experiments suggest that the N-terminal sequence is poorly accessible to the modifying enzyme until Aub binds a guide RNA, inducing a conformation change that exposes its N-terminus (Fig. 7a).

Structural differences between empty and loaded states were reported for several prokaryotic and eukaryotic Agos^16,71–77^, corroborating the idea that binding to guide RNA induces conformational change. The PAZ domain of Agos exhibit a high level of flexibility upon loading of guide RNA/DNA. During the recognition of target RNA, the PAZ domain undergoes a conformational transition that releases the 3′ end of the guide and facilitates downstream guide-target base pairing. Our results indicate that binding of the 3′ end by the PAZ domain is critical for sDMA modification of Aub’s N-terminal region. Unfortunately, the N-terminal extension region was often truncated to facilitate Ago expression and crystallization and thus reported structures do not provide information about the N-terminal extension region^27,45,46^. If the N-terminal region is preserved, it exists in an unstructured conformation that remains unresolved by crystallography^17,26,78^. However, piRNA loading of the nuclear PIWI protein in *Drosophila* was shown to induce a conformational change that exposes the nuclear localization sequence (NLS) located in its N-terminus and to enable its binding to importin^62^. Thus, two PIWI clade proteins, Aub and Piwi, harbor an N-terminal sequence that becomes accessible upon piRNA loading and its exposure promotes interactions with other factors and regulates protein function. Similar to Aub, the N-terminal extension region of Ago3 also harbors a (G/A)R motif that can be modified. Considering that piRNA binding triggers exposure of the N-terminus in Aub and Piwi, a similar process might occur in Ago3. Indeed, previous studies^42,47^ and our results revealed that, unlike the bulk of the cellular Ago3 pool, Krimper-bound Ago3 is both unloaded and unmethylated, indicating that piRNA binding and modification are correlated for Ago3 as well as for Aub.

Our results demonstrate the importance of the N-terminal region in the function of PIWI proteins. Unlike other domains (PAZ, MID, PIWI) of Argonautes with well-characterized functions in RNA guide binding and target cleavage, the N-terminal region has received little attention due to its disordered conformation and its low conservation between different Agos^17,79–83^. Our results suggest that the low conservation and absence of a fixed structure are in fact important features of the N-terminal region that are critical for PIWI proteins function. The flexible structure of this region might provide sensitivity to changes in overall protein conformation, such as the changes triggered by guide RNA binding. In Aub and Ago3, the modification and binding of sDMA sites to other proteins, as well as NLS-mediated interaction of Piwi require only a short linear motif, and thus the N-terminal region does not require a strongly conserved sequence or rigid folding. In agreement with this, the presence of a (G/A)R motif in Aub and Ago3 proteins is conserved in other *Drosophila* species, however, the specific position and sequence context of the motif is diverse (Fig. 1d, e). The poor similarity between N-terminal sequences of different Agos might endow them with distinct functions. It might be worth exploring whether signaling of the guide-loading state through exposure of the N-terminal region is also conserved in Ago-clade proteins and whether it regulates their function.

### Function of sDMA in the ping-pong cycle

The central feature of ping-pong is that the cleavage of target RNA by one PIWI protein results in the transfer of the cleaved product to another PIWI protein (Fig. 7b). Although the original model of ping-pong did not provide information on the molecular complex and interactions within the complex, ping-pong intuitively implies physical proximity between the two PIWI proteins followed by complex molecular rearrangements. We found that, although sDMA modification does not affect slicer activity of Aub (Supplementary Fig. 4d), information about the piRNA-loading state of PIWI proteins signaled by their sDMA modifications is used to assemble a complex that enables the transfer of the processed RNA from Aub to Ago3.

While our previous findings strongly suggest that Krimper plays a role in the assembly of the ping-pong piRNA processing (4 P) complex in which Aub and Ago3 are brought into close physical proximity^42^, the architecture of this complex and the extent to which Krimper regulates ping-pong remained unknown. Our results indicate that a single Krimper molecule interacts simultaneously with Aub and Ago3, suggesting that ping-pong takes place within a tertiary complex containing one molecule of each protein. Krimper actively selects the two ping-pong partners using the distinct specificities of its two Tudor domains: eTud1 uniquely binds Ago3, while eTud2 recognizes modified Aub (Fig. 1f and Supplementary Fig. 1a). We found that in vitro the eTud2 domain is capable of binding both sDMA-modified Aub and Ago3 peptides (Supplementary Fig. 1a), however, in vivo Krimper complexes were reported to contain exclusively unmodified Ago3^47^, suggesting that in the proper cellular context eTud2 only binds sDMA-Aub. Thus, the domain architecture of Krimper ensures that tertiary complexes contain Aub-Ago3 partners rather than random pairs. This finding is in line with the observation that ping-pong occurs predominantly between Aub and Ago3^2,5^, although, in principle, ping-pong can take place between two identical proteins, and a small level of homotypic Aub/Aub ping-pong was previously detected^3,84^. Thus, our results suggest that the propensity for heterotypic ping-pong is, at least in part, due to Krimper (Fig. 7a).

Ping-pong not only requires the physical proximity of two PIWI proteins but also that they have opposite piRNA-loading states: one protein induces piRNA-guided RNA cleavage (and therefore has to be loaded with a piRNA guide), while the other accepts the product of this reaction (and therefore has to be free of piRNA). Our results suggest that the opposite binding preference of the two Tudor domains towards sDMA ensures that the tertiary complex contains PIWI proteins in opposite RNA-loading states. While the overall fold structure of the two Tudor domains is similar, they have critical differences responsible for their distinct binding preferences. The binding pocket of eTud2 is similar to that of other Tudor domains and contains four aromatic residues that interact with sDMA (Fig. 2a, e). As sDMA modification of Aub signals its piRNA-binding status, the binding of eTud2 to modified Aub ensures that the complex contains Aub/piRNA. The structural studies and in vitro binding assays revealed that Ago3 binds to eTud1 in its unmethylated state and sDMA modification of any of the Arg residues within its (A/G)R motif prevents this interaction. The unusual binding preference of eTud1 is reflected in its non-canonical binding pocket, which lacks three of the four conserved aromatic residues (Fig. 2i). The binding of methylated Aub and unmethylated Ago3 ensures that Aub has a guide piRNA and Ago3 is free, thus enabling loading of Ago3 with RNA generated by Aub/piRNA-induced cleavage (Fig. 7a).

The architecture of the tertiary complex assembled by Krimper permits Aub-dependent generation and loading of RNA into Ago3. However, the ping-pong cycle also includes the opposite step, Ago3-dependent generation of Aub piRNA (henceforth we termed these steps “ping” and “pong”). Our results suggest that the ping and pong steps require the assembly of two distinct complexes discriminated by the modification status of Aub and Ago3.

### Formation of a membraneless cellular compartment and its function in ping-pong

As a single Krimper simultaneously binds Aub and Ago3, Krimper dimerization might be dispensable for ping-pong, raising the question of what the function of Krimper self-interaction is. Previous findings suggest that Krimper forms a scaffold for assembly of nuage, a membraneless organelle (MLO) that surrounds nuclei of nurse cells and resembles other MLO possibly formed through liquid–liquid phase separation^42^. Several lines of evidence point at Krimper as an essential component of nuage that acts as a scaffold for its assembly and the recruitment of client components. First, unlike other nuage components, FRAP measurements show very little Krimper exchange between nuage and the dispersed cytoplasmic compartment. Second, wild-type, but not mutant Krimper that lacks the self-interaction domain, forms cytoplasmic granules upon expression in heterologous cells that do not contain other nuage proteins. In contrast, other nuage components including Aub and Ago3 are dispersed in the cytoplasm when expressed in a similar setting, suggesting that they do not form condensates on their own and rely on other components for recruitment to nuage. Krimper recruits both Aub and Ago3 into MLO that it forms in heterologous cells. Combined, these data indicating that Krimper works as a scaffold, and Ago3 and Aub as its clients for nuage assembly. Thus, the interactions between Krimper and the N-terminal regions of Aub and Ago3 is not only essential for the assembly of the tertiary molecular complex but is also responsible for the recruitment of these proteins into membraneless cellular compartment (Fig. 7a). The high local concentration of proteins and RNA involved in the piRNA pathway in nuage might enhance the efficiency of ping-pong as well as the recognition of RNA targets by Aub and Ago3.

## Methods

### Fly stocks

Short hairpin RNA (shRNA) lines used for knockdown including sh-White (BDSC #33623) and sh-Zuc (BDSC #35227), maternal alpha-Tubulin 67C-Gal4 drivers on chromosome two (BDSC #7062) or chromosome three (BDSC #7063) in addition to the Aub mutant stocks aub^HN2^ cn^1^ bw^1^/CyO (BDSC #8517) and aub^QC42^ cn^1^ bw^1^/CyO, (BDSC # 4968) were obtained from the Bloomington *Drosophila* Stock Center. Flies were kept on yeast for 2 days and ovaries dissected 5 days after hatching.

### Generation of transgenic fly lines

Transgenic constructs for injection were generated using the Gateway cloning system (Life Technologies). cDNAs were obtained by RT-PCR from ovarian or testes RNA of adult *Drosophila melanogaster*, Oregon R strain. mdAub, pdAub, pdAub-EXT, and AubPAZmut were generated by overlap PCR and inserted into the pENTR-D-TOPO directional cloning vector (Life Technologies). Transgenes were cloned into the pUASP-Gateway-phiC31 fly injection vector derived from the pCasPeR5-phiC31 vector containing GFP, mKate2, or Strep-FLAG tags using the Gateway cloning system (Life Technologies). The expression of each transgene was controlled using the yeast upstream activation sequence promoter (UASp) stably crossed with a maternal a-Tubulin67c-Gal4-VP16 (MaG4) driver. Transgenes were generated in flies by PhiC31-mediated transformation (BestGene) using PhiC31 landing pads on either chromosome two (BDSC #9736) or chromosome three (BDSC #9750). The GFP-wtAub and GFP-mdAub BAC line was generated by cloning the *aub* genomic locus from the BAC clone BACN04M10 into the pCasPeR4 vector using restriction sites XhoI and SpeI. Bacterial recombineering (Gene Bridges Counter Selection kit) was used to insert an in-frame GFP tag in the start site of Aub. GFP-wtAub and GFP-mdAub rescue lines were generated by crossing transgenic construct into the aub^[HN]^/ aub^[QC]^ background.

### Cell culture, immunoprecipitation, and western blots

Schneider S2 cells were cultured in a complete Schneider medium (10% heat-inactivated FBS; 100U penicillin [Life technologies]; 100 μg streptomycin [Life technologies]). Plasmids were generated using Gateway cloning (Life technologies) using the *Drosophila* gateway vector collection (DGVC) destination vectors, pAGW, pAFW, and pAHW for GFP, 3xFLAG and 3XHA tags, respectively, driven by the Actin5C promoter. All constructs were N terminal tagged. Cells were transfected using TransIT-LT1 transfection reagent (Mirus biosciences) according to the manufacturer’s recommendation using 3 μg of total plasmid, with 1.5 μg each for double transfections, or 1.0 μg each for triple transfections. S2 cells were lysed in S2 lysis buffer (20 mM Tris at pH7.4, 200 mM KCl, 0.1% Tween-20,0.1% Igepal, EDTA-free Complete Protease Inhibitor Cocktail [Roche], 100 μg/mL RNase A). Supernatant was cleared by centrifugation at 4000×*g* for 20 min at 4 °C. Input sample was collected from the supernatant at concentrations of 1–3 μg/μL. Anti-FLAG M2 beads (Sigma Aldrich), anti-HA agarose beads (Thermo Fisher), and anti-GFP antibody (Covance) conjugated to Dynabeads (Thermo Fisher) were blocked in 5 mg/ml BSA for 10 min at 4 °C, followed by washing in S2 lysis buffer. Beads were added to the supernatant and rotated at 4 °C for 4 h, washed three times in lysis buffer and eluted by boiling in reducing SDS loading buffer. In the two-step co-IP, FLAG beads were eluted using 3X FLAG peptide (Sigma- Aldrich).

For co-IP from ovaries, dissected ovaries were lysed in NT2 buffer (50 mM Tris at pH 7.4, 150 mM NaCl, 1 mM MgCl_2_, 0.05% Igepal, EDTA-free Complete Protease Inhibitor Cocktail), while for IP experiments RIPA buffer (25 mM Tris at pH 7.4, 150 mM NaCl, 1% NP-40, 0.5 sodium deoxycholate and 0.1% SDS, Igepal, EDTA-free Complete Protease Inhibitor Cocktail) was used for lysis. Lysate was incubated in the presence or absence of 100 µg/mL RNase A and cleared by centrifugation. For co-IP experiments, lysates were incubated with anti-FLAG M2 beads (Sigma Aldrich) or with anti-GFP antibody (Covance) conjugated to Dynabeads (Thermo Fisher) at 4 °C for 4 h. For IP experiments, lysates were incubated with GFP nanobody (Chromo Tek), followed by washing and elution in reducing SDS buffer. Western blot was probed with rabbit anti-GFP (Covance) (1:3 K), rabbit anti-Zuc (Endogenous) (1:1 K), rabbit SYM11 antibody (Sigma Aldrich) (1:1 K), mouse anti-GFP (Santa Cruz) (1:3 K), anti-FLAG M2 (Sigma Aldrich) (1:10 K), mouse anti-HA (Sigma Aldrich) (1:10 K).

### piRNA isolation from immunopurified protein complexes

Immunopurified protein-RNA complexes were spiked with 5 pmol of 42-nt RNA oligonucleotide, followed by proteinase K digestion and phenol extraction. Isolated RNA was CIP treated, radiolabeled using PNK and gamma-P32 ATP, and run on a 15% urea-PAGE gel. Semi-Quantitative piRNA binding analysis and a detailed protocol can be found in a previous paper^42^.

### Small RNA-seq

Small RNA libraries were cloned from the total ovarian lysate of Aub heterozygous, aub^[HN]^/ aub^[QC]^ mutant and aub^[HN]^/ aub^[QC]^, GFP-wtAub or GFP-mdAub rescue flies. 30 ovaries were dissected, and total RNA was isolated with Ribozol (Amresco, N580). Small RNAs within a 19- to 29-nt window were isolated from 15% polyacrylamide gels from 4 µg of ovarian total RNA. For samples that were NaIO_4_-treated, 5× borate buffer (pH 8.6; 150 mM borax, 150 mM boric acid) and 200 mM sodium periodate were added to the size-selected small RNA, and the samples were incubated for 30 min at 25 °C. The NaIO_4_-treated small RNA was then ethanol-precipitated before proceeding to library construction. The small RNA libraries were constructed using the NEBNext small RNA library preparation set for Illumina (no. E7330S), according to the protocol, using NEBNext multiplex oligos for Illumina (no. E7335S). Libraries were sequenced on the Illumina HiSeq 2500 (SE 50-bp reads) platform.

### Immunoprecipitation small RNA-seq

Ovaries (∼100 per immunoprecipitation) from flies expressing GFP-wtAub and GFP-mdAub under the control of endogenous promoter were dissected and lysed on ice in 250 µL of lysis buffer [30 mM Hepes-KOH at pH7.4, 100 mM KOAc, 2 mM Mg(OAc)_2_, 5 mM DTT, 0.5% (v/v) NP40, proteinase inhibitor (Roche, 11836170001), RNasin Plus (Promega, N2611)]. Lysate was dounced and clarified by centrifugation at maximum speed at 4 °C. The supernatant was incubated with rabbit polyclonal anti-GFP (Covance) conjugated to Dynabeads (Thermo Fisher) for 4 h at 4 °C. 10% of immunoprecipitate was used for western blotting, while the rest was used to isolate RNA. Five 5 pmol of 42 nt RNA oligonucleotide (42 M) was added to the beads to assess purification efficiency followed by proteinase K digestion and phenol extraction of RNA. A fifth of the RNA was CIP-treated (New England Biolabs, M0290S) in NEB buffer #3 (New England Biolabs, B7003S) for 30 min at 37 °C and then ethanol-precipitated after phenol:chloroform and chloroform extraction. The CIP-treated RNA was then PNK-treated with 1 µL of 10× T4 polynucleotide kinase buffer (New England Biolabs, B0201S), 2 µL of [γ-P^32^] ATP (PerkinElmer, BLU502A250UC), and 1 µL of T4 polynucleotide kinase (New England Biolabs, M0201S) for 45 min at 37 °C. The CIP- and PNK-treated RNA was added back to the remainder of the RNA isolated from the immunoprecipitation. Size selection, library preparation, and analysis were performed as described in small RNA-seq, except that fragments were gel-extracted based on labeled immunoprecipitation material.

### Sequence analysis of piRNA libraries

After adapter trimming, reads were mapped to the dm3 assembly of the *Drosophila melanogaster* genome using Bowtie 0.12.7^85^. Only reads that map to the genome with no mismatches were considered for further analysis. piRNAs were defined as 23–29 nt reads that mapped to the repeat masker track (http://www.girinst.org/), while miRNAs were defined based on their mapping to annotated miRNA genes

To analyze piRNA generation throughout the genome, the genome was split into 5 kb intervals and windows that produce at least 5 reads per million of miRNAs in control (*aub* heterozygous) and showed more than 80% reduction of piRNAs in the *aub* mutants were further analyzed. To compare piRNA abundance between the libraries piRNA reads were normalized to total miRNA reads. For a detailed comparison of piRNA generation from 42AB, 38 C, 80EF, and *flamenco* clusters, 1 kb genomic intervals were used.

To analyze piRNAs to individual TE families piRNA reads were mapped to TE consensus sequences (http://www.fruitfly.org/p_disrupt/TE.html) allowing for up to 2 mismatches. To compare TE piRNA abundance between the libraries piRNA reads were normalized to total miRNA reads. The fold-change in read count for each TE family was calculated by obtaining the base 2 logarithm of ratio of normalized reads in experimental libraries and heterozygous control libraries. To obtain the fraction of ping-pong piRNA pairs for each TE family, we measure the likelihood that a piRNA has a “partner” with 10 nt overlap. We first matched all piRNA sequences to the Repbase transposon sequences, allowing for plus and minus strand matches with up to 2 mismatches, indels, or bulges. Each piRNA was reduced to its 5′ end. For each matched piRNA (“target piRNA”), we computed a histogram of distances/offsets to “partner” piRNAs that match to the opposite strand, for which each partner piRNA contributes its cloning/sequencing count, normalized to 100% overall partner piRNAs up to a distance of 29 nt (note that each piRNA is a “partner” piRNA for all other piRNAs and also a “target” piRNA itself with all other piRNAs as “partners”). We then combined these histograms for each distance/offset by summing the values for that distance over all histograms but weighting each histogram’s contribution by the relative cloning/sequencing of the respective target piRNA for which the histogram was computed. To compare the abundance of TE piRNA associated with wtAub- and mdAub, reads mapping to each TE family were normalized to total library reads (RPM) after rRNA reads were discarded.

### Microscopy

Ovaries were dissected in PBS and fixed in 4% PFA in PBS for 20 min, washed 3 × 10 min at RT, permeabilized in 1% Triton-X100 in PBS for 10 min, and DAPI stained (Sigma-Aldrich). Ovaries were washed in PBS and mounted in Vectashield medium (Vector Labs). S2 cells were allowed to settle on coverslips treated with Poly-L-Lysine (Sigma-Aldrich). After gentle washing, cells were fixed in 0.5% PFA in PBS for 20 min followed by staining with DAPI (Sigma-Aldrich), washed, and mounted in Vectashield medium (VectorLabs). Images were captured using an AxioImager microscope; an Apotome structured illumination system was used for optical sections (Carl Zeiss).

### In vitro cleavage assay

The in vitro assay for Aub slicer activity was adapted from a method described previously^5^. FLAG-tagged wild-type and mdAub proteins were expressed and immunopurified from S2 cells. Proteins were eluted using 3X Flag peptide (Thermo Fisher). In total, 50 K MWCO protein concentrator (Thermo Fisher) was used to remove the 3X Flag peptide. In total, 100 nM purified proteins were incubated with 100 nM of single-strand 26 nt guide RNA in cleavage buffer (25 mM Hepes-KOH, ph 7.5, 50 mM KOAc, 5 mM Mg(OAc)_2_, 5 mM DTT) for 90 min at 25 °C, and then ^32^P-5′-labeled 29 nt complementary RNA (PerkinElmer, BLU502A250UC) was added and incubated for another 90 min. The cleaved products were analyzed on urea-containing PAGE.

### Limited protease assay

In total, 10 ng/µl chymotrypsin stock solution was prepared in chymotrypsin reaction buffer (10 mM Tris-HCl [pH 8.0], 2 mM CaCl_2_, and 5% glycerol). FLAG-wtAub was expressed in S2 cells. Lysates were divided into two equal fractions, one was incubated with 26 nt ssRNA oligo for 1 h at RT, another was incubated without oligos, followed by immunoprecipitation using anti-FLAG M2 beads. Beads were washed three times with lysis buffer and then incubated with a 1:2 K, 1:4 K, and 1:8 K serial dilution of the thermolysin protease for 30 min at 37 °C. After extensive washing, beads were eluted in reducing SDS buffer. Samples were analyzed by western blot using an anti-FLAF M2 antibody.

### Protein disorder prediction and conservation analysis

Disorder predictions of full-length Krimper and the N terminal region of Aub and Ago3 were obtained using the PrDOS server based on a previously used algorithm^86^. Conservation is measured as a numerical index reflecting the conservation of physico-chemical properties of amino acids in the alignment: Identities score highest, followed by substitutions to amino acids lying in the same physico-chemical class. A detailed description of the algorithm can be found in^87^.

### Hatching rate calculation

Three-day-old mated adult female flies fed on yeast paste were transferred to fresh grape agar plates and allowed to lay eggs for 12 h. Eggs were counted, and the hatching rate was determined over the following 36 h. Counting was repeated on ten consecutive days.

### RT-qPCR

Total RNA was isolated from 20 ovaries with Ribozol (Amresco, N580). RNA was DNase I treated (Invitrogen, 18068-015) and reverse transcribed using SuperScript III (Invitrogen) with oligo d(T)_15_ according to the manufacturer’s recommendations. RT-qPCR was performed using Mastercycler ep Realplex PCR thermal cycler machine (Eppendorf). Target expression was normalized to *rp49*. Primers are shown in Table S2.

### Fluorescence recovery after photobleaching (FRAP)

For each construct, at least 30 independent FRAP experiments were performed using ovaries expressing a single GFP-tagged transgene under the endogenous promoter. FRAP experiments were captured on a Zeiss LSM710 confocal microscope (Carl Zeiss AIM) equipped with a 25×/0.8 NA Imm. Corr. Multi-immersion objective lens using the Zeiss Zen Black software. Image acquisition for all experiments utilized an identical 488 nm AOTF laser power setting of 7% to ensure laser power was not influencing measurements. PMT gain settings were variably set to accommodate the expression level of each GFP tagged protein. Images were acquired at 256 × 256-pixel resolution at 0.07 μm pixel size and scan speed of 614.4 ms per frame with 1.0 μs pixel dwell time. A single bleach region was defined for each experiment, consisting of a region of 7 × 7 pixels equal to 0.49 μm × 0.49 μm and was bleached by a single iteration of 100% laser power from 488, 561 and 633 nm wavelengths. Five initial pre-bleach images were captured prior to bleaching and 115 subsequent post bleach images were acquired every 614.4 ms to assess fluorescence recovery in the bleach zone. FIJI (FIJI; http://fiji.sc/) software was used to analyze FRAP experiments. To account for background and photo-bleaching effects during acquisition, the mean intensity values from the bleach zone (BL), the background zone (BG), and the reference signal zone (REF) were used to calculate the corrected BL(BLcorr) for each acquisition frame using the equation:

$${\rm{BLcorr}}=\frac{[{\rm{BL}}-{\rm{BG}}]}{[{\rm{REF}}-{\rm{BG}}]}$$

BLcorr values were normalized to the mean of five pre-bleach values, which were used to estimate 100% fluorescence intensity. Using the curve fitter module of FIJI, the normalized post-bleach data were fit to an exponential recovery model:

$$y=a\left(1-{e}^{-{bx}}\right)+{\rm{c}}$$

The mobile fraction was obtained by the sum of coefficients *a* and *c*, which describes the maximal extent of recovery for each experiment.

### Nuage:cytoplasm localization analysis

The occupancy of GFP-tagged protein in either nuage versus cytoplasm was calculated by obtaining the ratio of mean signal intensities of a perinuclear nuage area (*N*); an adjacent cytoplasmic area (*C*) of the equal area; and a background area (*G*) using the following equation:

$${N:C}=\frac{[{\rm{N}}-{\rm{G}}]}{[{\rm{C}}-{\rm{G}}]}$$

All images were acquired from the fixed specimen using the Apotome structured illumination system and a 40× oil-immersion objective.

### RNA binding in cell lysates and measurement of methylation level

S2 cells were transfected with 10 μg of a plasmid expressing N terminally Flag tagged wtAub driven by the Actin5C promoter. After 48 h incubation, cells were lysed in S2 lysis buffer (20 mM Tris at pH7.4, 200 mM KCl, 0.1% Tween-20,0.1% Igepal, EDTA-free Complete Protease Inhibitor Cocktail [Roche], 100 μg/mL RNase A). Lysates were divided into four equal fractions. In total, 26 nt synthetic RNA oligo (IDT) labeled with [γ-P^32^] ATP (PerkinElmer, BLU502A250UC) as described above was added into lysate fractions to a final concentration 1 μM. Lysates were incubated at RT for 1 h followed by IP with FLAG M2 beads at 4 °C for 4 h. Half of the beads were subject to RNA isolation, the other half was used for western blot. In the oligo binding concentration gradient experiment, lysates were divided into four equal fractions. non-radioactivity labeled 5′-end phosphorylated ssRNA oligo (IDT) was added into each fraction to a final concentration of 0, 0.1, 0.5, and 1 μM, respectively. Lysates were incubated at RT for 1 h followed by anti-FLAG IP at 4 °C for 4 h. Protein and methylation levels were detected by Western blot and methylation level was estimated as the ratio of the background-subtracted methylation signal intensity and the background-subtracted protein signal intensity.

Relative methylation level was measured by normalizing to the methylation level of the no oligo control.

### Protein expression and purification

Krimper eTud1 (residues 272-512) was cloned into a self-modified pSumo vector with 10×His tag followed by a yeast sumo sequence. The plasmid was transformed into *E. coli* strain BL21(DE3) Rosseta and cultured at 37 °C in LB medium. The protein expression was induced by adding IPTG to a final concentration of 0.2 mM when the OD_600_ reached 0.7, and the cells were cooled to 16 °C. The recombinant expressed protein was purified using a HisTrap column (GE Healthcare). The hexahistidine plus yeast sumo tag was removed by ulp1 protease digestion followed by a second step HisTrap column (GE Healthcare). The target protein was further purified using MonoQ and Superdex G75 columns (GE Healthcare).

Krimper eTud2 (residues 562-746) was cloned into a self-modified His-MBP vector. Protein production procedure was to with Krimper eTud1. The recombinant expressed protein was purified using a HisTrap column (GE Healthcare). The hexahistidine plus MBP tag was removed by TEV protease digestion followed by a second step HisTrap column (GE Healthcare). Protein was further purified using Q and Superdex G75 columns (GE Healthcare).

### Crystallization and crystal growth

Krimper eTud1 (272-512) was concentrated to 20 mg/ml and screened in sitting drop at 4 °C. Crystals were grown for 5 days in 0.1 M HEPES pH 7.5, 1.26 M (NH4)_2_SO_4_. For obtaining the protein-peptide complex, 20 mg/ml Krimper eTud1 was mixed with Ago3-2 peptide with a molar ratio of 1:4 and incubated for 1 h before setting up sitting drop screening at 4 °C. Crystals were grown for 5 days in 0.1 M HEPES pH 7.5, 1.5 Li_2_SO_4_. 10 mg/ml Krimper eTud2 was mixed with AubR15me2 peptide with a molar ratio of 1:4 and incubated for 1 h before sitting drop. Crystals appeared in 2 days in 16% PEG3350 and 0.1 M NH4Ac.

### Data collection and structure determination

All data were collected at the Shanghai Synchrotron Radiation Facilities beamline 18U1 (SSRF-BL18U1) and beamline 19U1 (SSRF-BL19U1). Data were processed using the program HKL3000^88^. Structures of eTud1-Ago3 complex and eTud2-Aub complex were solved by SAD using the anomalous signals of SeMet using the program Phenix^89^. eTud1 apo structure was solved by molecular replacement using the program Phenix^89^ with eTud1-Ago3 complex as a search model. Models were refined in Phenix^89^ and COOT^90^ iteratively and finally presented using Pymol (DeLano Scientific).

### ITC

All ITC was performed using the Malvern PEAQ ITC instrument (Malvern) in a buffer of 50 mM NaCl, 20 mM HEPES pH 7.5, and 2 mM β-mercaptoethanol. Data analysis was performed using the Malvern data analysis software and Origin 7.0.

### Reporting summary

Further information on experimental design is available in the Nature Research Reporting Summary linked to this paper.

## Supplementary information

Supplementary file

Peer Review File

Reporting summary

## Data Availability

All relevant data supporting the key findings of this study are available within the article and its Supplementary Information files or from the corresponding authors upon reasonable request. The *Drosophila* strains that we have used in this report are also available from the corresponding authors upon reasonable request. Source data are provided with this paper. Small RNA-seq data are available on the GEO database, GSE153156. X-ray structures have been deposited in the RCSB Protein Data Bank with the accession codes: 7CFB for the eTud1 apo structure, 7CFC for the eTud1-Ago3 complex structure, and 7CFD for the eTud2-AubR15me2 structure. Source data are provided with this paper.

## Source data

source data
