# Supplementary material for: Binding of guide piRNA triggers methylation of the unstructured N-terminal region of Aub leading to assembly of the piRNA amplification complex
Source: Nat Commun. 2021 Jul 1;12:4061. doi: 10.1038/s41467-021-24351-x (PMC8249470; doi:10.1038/s41467-021-24351-x)

Binding of guide piRNA triggers methylation of the unstructured N-terminal region of Aub leading to assembly of the piRNA amplification complex

Supplementary Figures

Supplementary Figure 1. Interaction between Krimper, Aub and Ago3.

(a) eTud2 preferentially binds methylated peptides. ITC analysis of Krimper eTud2 interaction with different Ago3 or Aub peptides.

(b) Co-immunoprecipitation of Aub with Krimper fragments in S2 cells. FLAG-tagged eTud1 and N+eTud1 fragments were coexpressed with HA-Aub in S2 cells. Anti-FLAG immunoprecipitation was followed by Western blot. Unlike the N+eTud1 fragment, the eTud1 fragment without the N-terminal domain does not co-IP with Aub. Experiment reproduced twice with similar results.

Supplementary Figure 2. Detailed analysis of Krimper eTud1 apo structure.

(a) Detailed interactions of eTud2 with Aub peptide shown in yellow. Hydrogen bonds are shown in grey dashed lines.

(b) Overall structure of the Krimper eTud1 domain. The ‘latch helix’ region is highlighted in magenta.

(c) Detailed interactions of the ‘latch helix’ shown in magenta with other parts of Krimper eTud1.

(d) Overall structure of the Krimper eTud1-Ago3-2 complex, with Ago3-2 shown in orange.

(e) Structural superposition of Krimper eTud1 apo (magenta) and Ago3 peptide-bound structure (orange).

(f) Detailed interaction networks of Krimper eTud1 with the Ago3-2 peptide shown in orange.

(g) The Phe400 side chain in the Ago3 peptide bound structure (green) rotates 90° to accommodate Ago3-R70 when compared with the apo structure (magenta).

Supplementary Figure 3. Structural comparison of Krimper eTud1-Ago3-2 complex with other extended Tudor domains.

(a) Electrostatic surface of the TDRD2-PIWIL1 complex binding cleft (PDB code:6B57 [http:// 10.2210/pdb6B57/pdb]). With PIWIL1 peptide shown in yellow.

(b) Sequence alignment between TDRD2 and eTud1 of Krimper. The position of amino acids involved in the hydrophilic contacts in the TDRD2-PIWIL1 structure are labeled with red arrows above the alignment. The position of amino acids involved in the hydrophobic contacts in the TDRD2-PIWIL1 structure are labeled with blue arrows above the alignment. The position of amino acids involved in the hydrophilic contacts in the eTud1-Ago3 structure are labeled with red arrows below the alignment. The position of amino acids involve in the hydrophobic contacts in the eTud1-Ago3 structure are labeled with blue arrows below the alignment. The same amino acids between two proteins are colored in red, the similar amino acids are colored in yellow.

(c) eTud1 N-terminal structure is highlighted in brown.

(d) Structural superposition of eTud1 apo structure colored in green with a canonical extended Tudor domain (SND1-PIWIL1 colored in cyan, PDB code: 3OMC [http://10.2210/pdb3OMC/pdb]). The position of the linker helix is different in the two structures.

Supplementary Figure 4. Localization, mobility and slicer activity of wtAub and mdAub proteins.

(a) Expression of EGFP-tagged wtAub, mdAub and pdAub proteins. Lysates were prepared from 100 ovaries dissected from flies expressing EGFP-tagged wtAub, mdAub and pdAub proteins and 3% of each lysate was loaded on the gel followed by Western blot analysis using anti-EGFP antibody. Experiment reproduced twice with similar results.

(b) Localization of GFP-wtAub and GFP-mdAub expressed under the control of

the endogenous Aub promoter in nurse cells at stage 6 of oogenesis. Scale bar: 5μm. Eighty nurse cells within ten independent ovaries were used for imagining with similar results.

(c) Methylation deficiency increases Aub mobile fraction. Representative FRAP experiments showing that the normalized recovery of GFP-mdAub is approximately 10% greater compared to GFP-wtAub. The mobile fraction was determined by modeling the recovery to an exponential recovery curve. Thirty nurse cells within ten independent ovaries were used for imagining with similar results.

(d) mdAub has slicer activity. Purified wtAub and mdAub were used in a cleavage assay with 29 nt 5' end radiolabeled target containing a sequence complementary to the guide RNA. The products were resolved on a denaturing urea gel. Western blot shows the amounts of wtAub and mdAub used in the cleavage assay. Experiment reproduced twice with similar results.

Supplementary Figure 5. Analysis of piRNAs in flies expressing wtAub and mdAub proteins.

(a) Nucleotide biases in piRNA populations in ovaries of wtAub- and mdAub-rescue flies. Total 18 to 30 nt RNA was cloned from ovaries with the indicated genotypes and piRNAs were defined as 23 to 29 reads that mapped to the repeat track of the genome in sense and antisense orientation relative to the annotated TE sequence. The graphs show frequencies of 1U and 10A in the sense and anti-sense piRNAs in libraries cloned from control (aub heterozygous), wtAub and mdAub rescues and aub mutant ovaries.

(b) Length distribution of small RNAs bound by GFP-wtAub and GFP-mdAub in the wildtype aub background.

Supplementary Figure 6. Oligo binding causes Aub conformational change

(a) Scheme of limited protease assay (top). S2 cell lysate expressing FLAG-

Aub was incubated with or without 1μM synthetic ssRNA oligos followed by FLAG IP. Bead-bound proteins were incubated with different concentration of chymotrypsin. Bead fraction was analyzed by Western blot (bottom) using FLAG detection. Red arrow indicates FLAG-tagged N-terminal fragment that is undigested in the absence of piRNA loading but digested upon small RNA loading. Experiment reproduced twice with similar results.

(b) CsuI interacts with the N-terminus of Aub. FLAG tagged CsuI was co-expressed in S2 cells with different GFP-tagged Aub fragments and coIP followed by Western detection of IP-ed proteins. Asterisk indicates bands corresponding to indicated GFP-tagged Aub protein fragments. Experiment reproduced twice with similar results.

Supplementary Tables:

Supplementary Table 1. Structural Data collection and refinement statistics

	Krimper Tud1 apo	Krimper Tud1-Ago3	Krimper Tud2-AubR15me2
**Data collection**			
Beamline	SSRF-BL19U1	SSRF-BL19U1	SSRF-BL19U1
Space group	$P2_12_12_1$	$P3_1$	$P2_12_12_1$
Wavelength (Å)	0.9792	0.9792	0.9792
Cell dimensions (Å)			
a	60.5	88.5	97.1
b	87.7	88.5	101.4
c	90.2	181.7	191.3
Resolution (Å)	50.0-2.1 (2.18-2.10) [a]	50.0-2.4 (2.49-2.40)	50.0-2.7 (2.80-2.70)
R_{merge}	0.134 (1.029)	0.094 (0.891)	0.114 (0.511)
$I / \sigma I$	12.5 (1.4)	9.0 (1.25)	12.5 (1.53)
Completeness (%)	99.8 (100.0)	99.3 (98.6)	95.5 (75.1)
Redundancy	5.4 (5.5)	4.3 (4.3)	5.1 (3.7)
CC1/2	0.727	0.872	0.897
**Refinement**			
R_{work} / R_{free}	0.204 / 0.222	0.234 / 0.259	0.243 / 0.291
No. reflections	28,403	61,721	49,822
No. atoms	3,274	7,519	12,195
Protein	3,062	6,899	11,794
Peptide	-	325	327
Solvent	212	295	74
B-factors (Å^2)	39.3	56.4	58.1
Protein	39.1	56.0	57.9
Peptide	-	67.6	69.2
Solvent	42.8	53.3	50.7
R.m.s. deviations			
Bond lengths (Å)	0.023	0.004	0.009
Bond angles (°)	1.771	0.743	1.237

[a] Highest-resolution shell is shown in parentheses.

Supplementary Table 2. Oligo and primer sequences

qPCR primers

HeT-A-F	CGCGCGGAACCCATCTTCAGA
HeT-A-R	CGCCGCAGTCGTTTGGTGAGT
ZAM-F	ACTTGACCTGGATACACTCACAAC
ZAM-R	GAGTATTACGGCGACTAGGGATAC
Burdock-F	AGGGAAATATTTGGCCATCC
Burdock-R	TTTTGGCCCTGTAAACCTTG
TAHRE-F	CTGTTGCACAAAGCCAAGAA
TAHRE-R	GTTGGTAATGTTCGCGTCCT
RP49-F	CCGCTTCAAGGGACAGTATCT
RP49-R	ATCTCGCCGCAGTAAACG

RNA oligos

13nt ss RNA (size marker)	rCrCrArUrCrGrArUrArArArArG
26nt ssRNA	rUrCrGrArArGrUrArUrUrCrCrGrCrGrUrArCrGrUrGrArUrGrUrU
29nt target ssRNA	rArCrCrArArCrArUrCrArCrGrUrArCrGrCrGrGrArArUrArCrUrUrCrGrA
30nt ssRNA (size marker)	rCrCrArUrCrGrArUrArArArArGrUrUrUrArArArCrGrArGrCrUrUrCrCrCrG
42nt ssRNA (size marker)	rCrCrArUrCrCrArUrCrGrArUrArArArArGrUrUrUrArArArCrGrArGrCrUrUrCrCrCrGrCrGrUrArCrGrGrA

Supplementary Figure 1

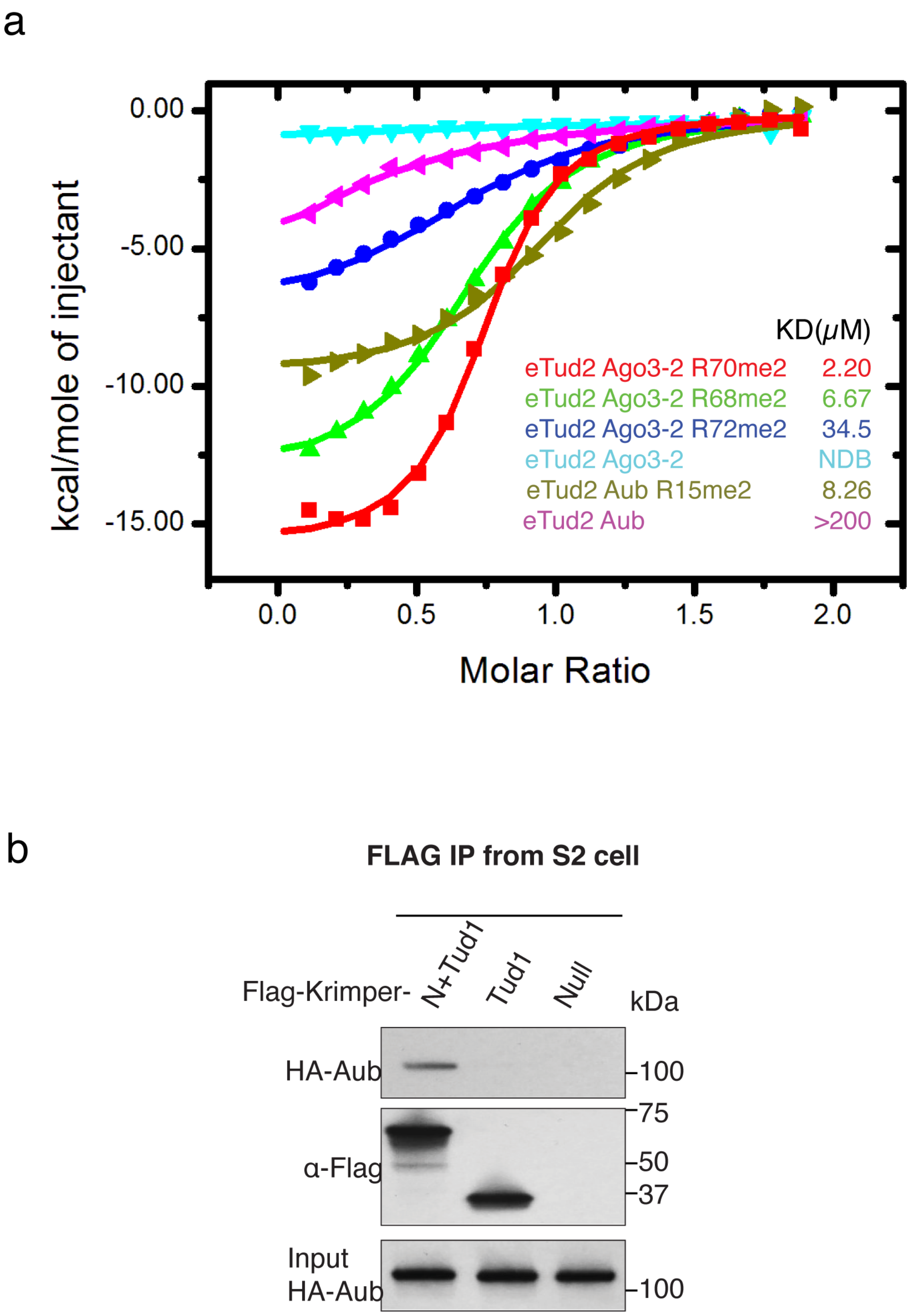

Supplementary Figure 2

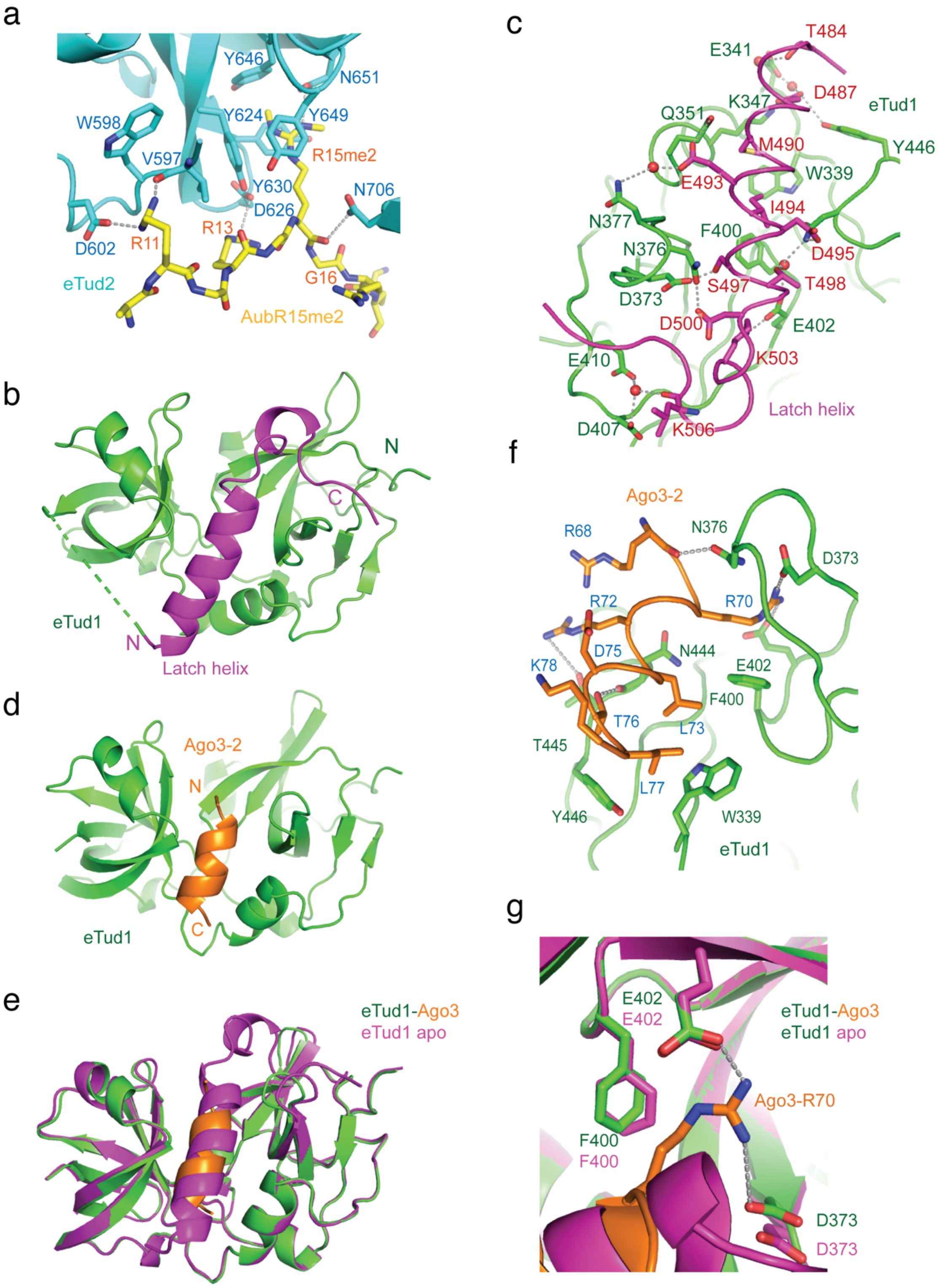

Supplementary Figure 3

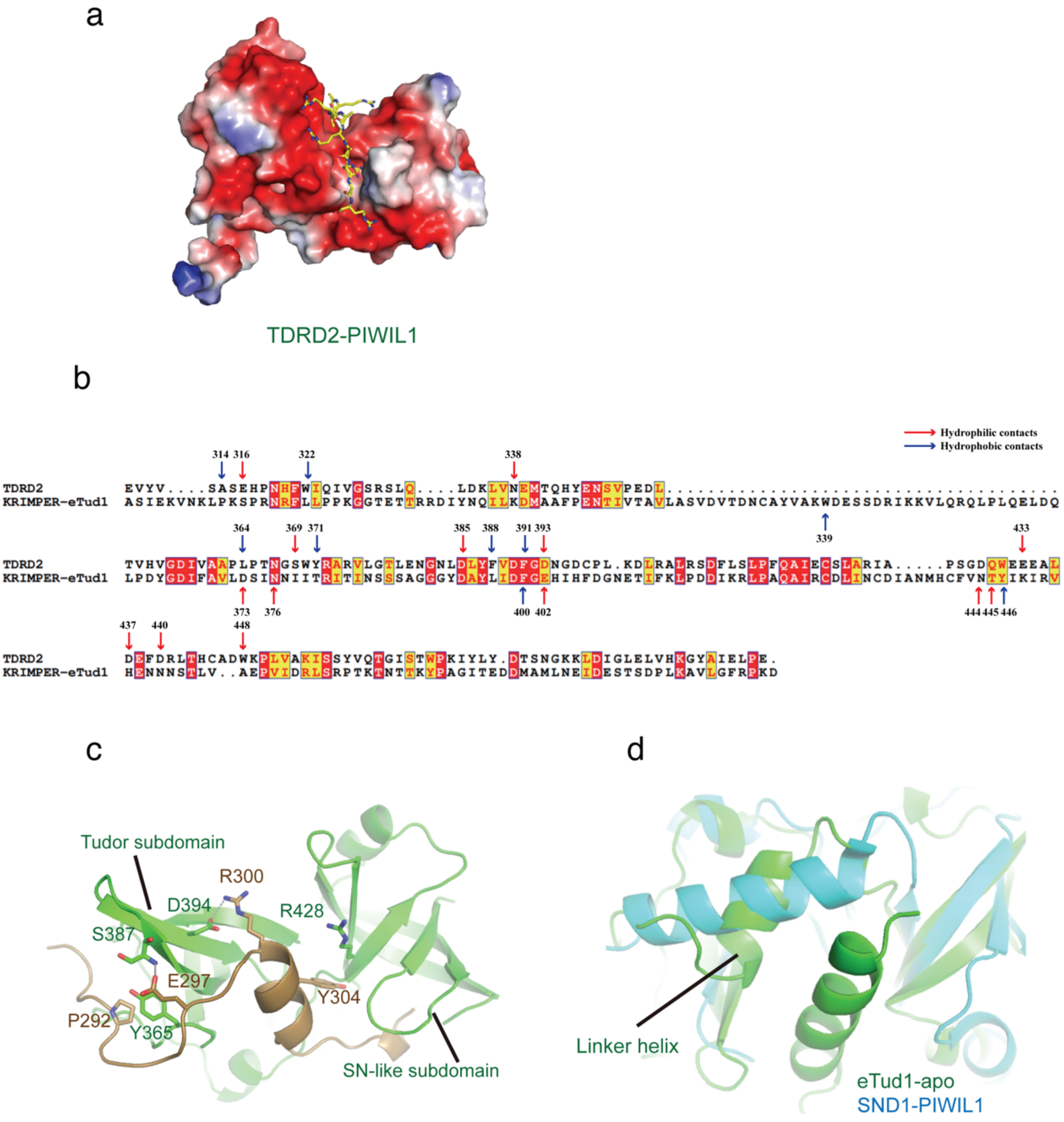

Supplementary Figure 4

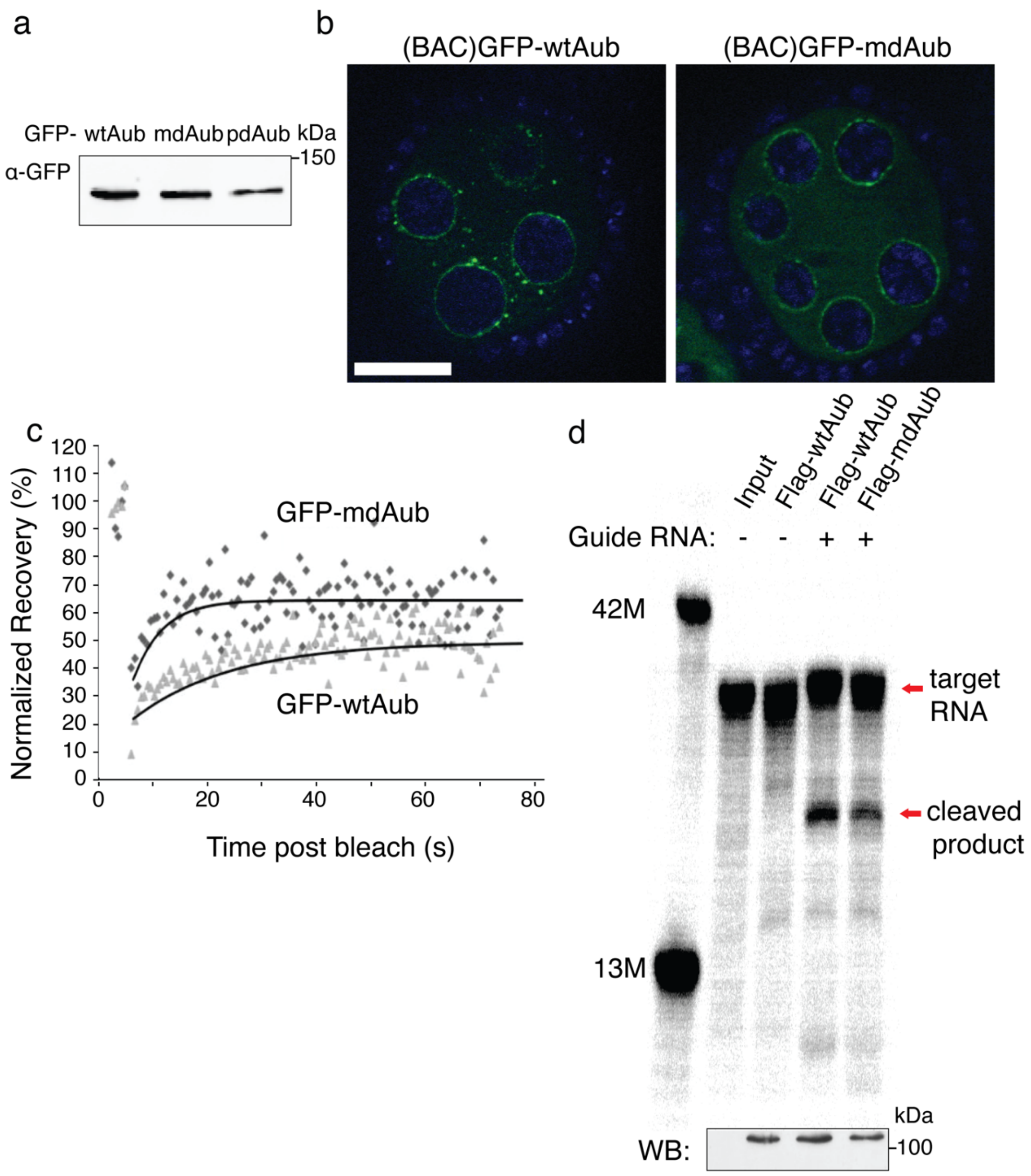

Supplementary Figure 5

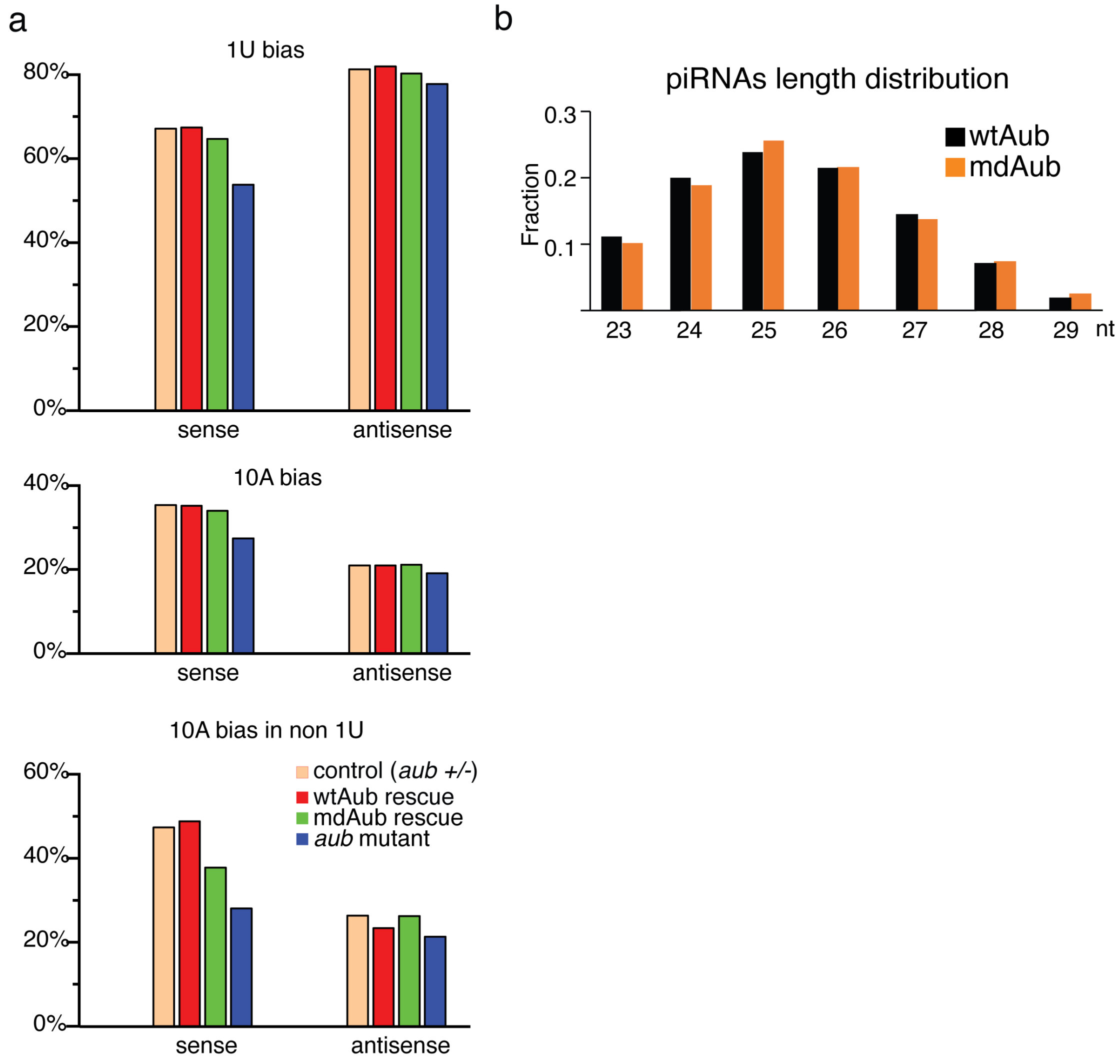

Supplementary Figure 6

a

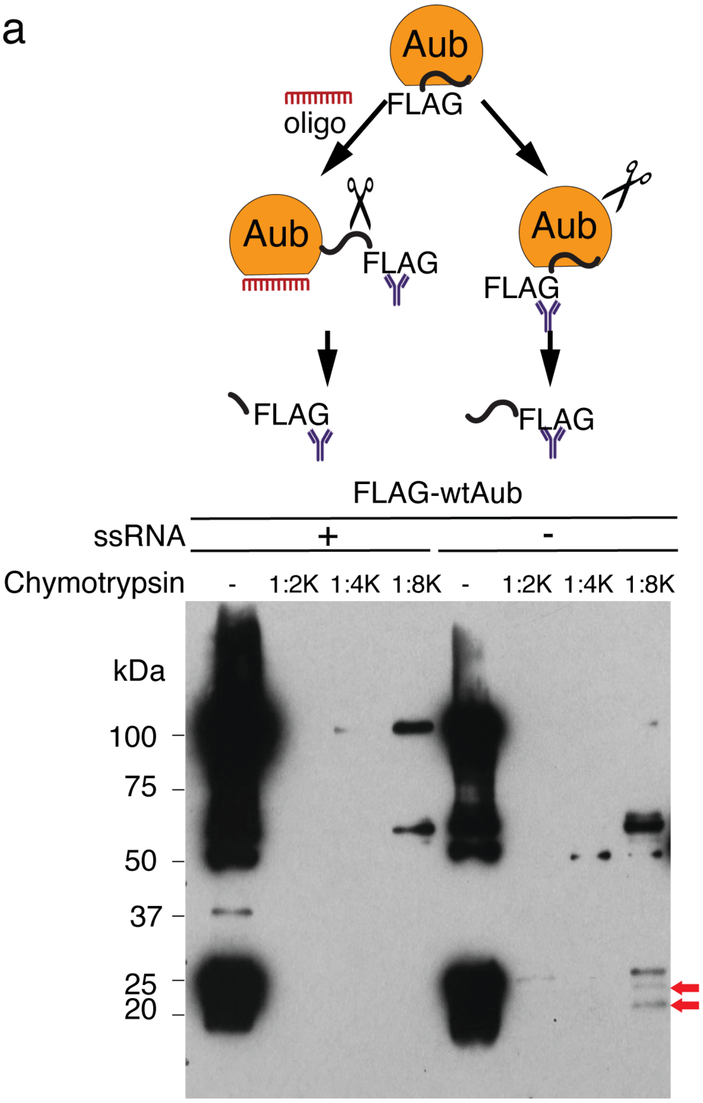

b

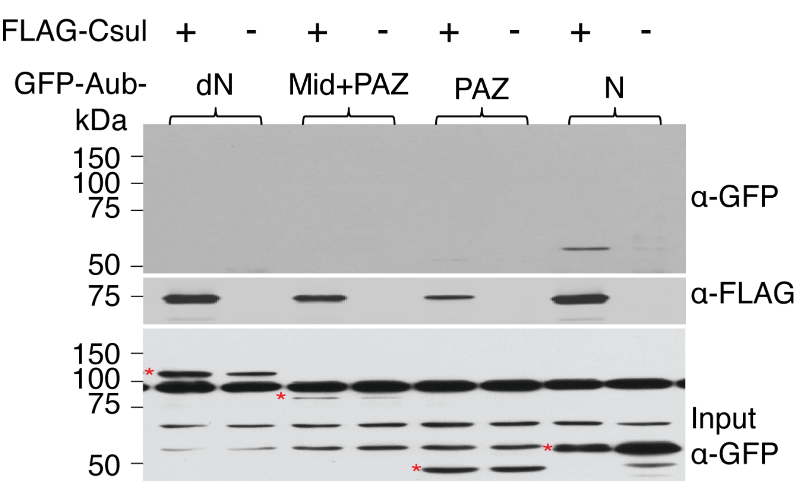

Supplement: Supplementary file 1 — Supplementary file [file 41467_2021_24351_MOESM1_ESM.pdf]
