# Supplementary material for: Binding of guide piRNA triggers methylation of the unstructured N-terminal region of Aub leading to assembly of the piRNA amplification complex
Source: Nat Commun. 2021 Jul 1;12:4061. doi: 10.1038/s41467-021-24351-x (PMC8249470; doi:10.1038/s41467-021-24351-x)
Supplement: Supplementary file 3 — Reporting summary [file 41467_2021_24351_MOESM3_ESM.pdf]

nature research

Corresponding author(s): Sisi Li & Alexei A. Aravin

Last updated by author(s): Oct 28, 2020

Reporting Summary

Nature Research wishes to improve the reproducibility of the work that we publish. This form provides structure for consistency and transparency in reporting. For further information on Nature Research policies, see our Editorial Policies and the Editorial Policy Checklist.

Statistics

For all statistical analyses, confirm that the following items are present in the figure legend, table legend, main text, or Methods section.

n/a	Confirmed	
☐	☒	The exact sample size (*n*) for each experimental group/condition, given as a discrete number and unit of measurement
☐	☒	A statement on whether measurements were taken from distinct samples or whether the same sample was measured repeatedly
☐	☒	The statistical test(s) used AND whether they are one- or two-sided<br>*Only common tests should be described solely by name; describe more complex techniques in the Methods section.*
☒	☐	A description of all covariates tested
☐	☒	A description of any assumptions or corrections, such as tests of normality and adjustment for multiple comparisons
☐	☒	A full description of the statistical parameters including central tendency (e.g. means) or other basic estimates (e.g. regression coefficient) AND variation (e.g. standard deviation) or associated estimates of uncertainty (e.g. confidence intervals)
☒	☐	For null hypothesis testing, the test statistic (e.g. *F*, *t*, *r*) with confidence intervals, effect sizes, degrees of freedom and *P* value noted<br>*Give P values as exact values whenever suitable.*
☒	☐	For Bayesian analysis, information on the choice of priors and Markov chain Monte Carlo settings
☒	☐	For hierarchical and complex designs, identification of the appropriate level for tests and full reporting of outcomes
☐	☒	Estimates of effect sizes (e.g. Cohen's *d*, Pearson's *r*), indicating how they were calculated

Our web collection on statistics for biologists contains articles on many of the points above.

Software and code

Policy information about availability of computer code

Data collection	HKL2000/3000, X-ray data collection and processing;
Data analysis	Coot, model building; Phenix, structure refinement; Molprobity, structure geometry analysis;

For manuscripts utilizing custom algorithms or software that are central to the research but not yet described in published literature, software must be made available to editors and reviewers. We strongly encourage code deposition in a community repository (e.g. GitHub). See the Nature Research guidelines for submitting code & software for further information.

Data

Policy information about availability of data

All manuscripts must include a data availability statement. This statement should provide the following information, where applicable:

- Accession codes, unique identifiers, or web links for publicly available datasets
- A list of figures that have associated raw data
- A description of any restrictions on data availability

High-throughput sequencing data for small RNA-seq experiments are available through Gene Expression Omnibus GSE153156; X-ray structures have been deposited in the deposited in the RCSB Protein Data Bank with the accession codes: 7CFB for the eTud1 apo structure, 7CFC for the eTud1-Ago3 complex structure and 7CFD for the eTud2-AubR15me2 structure

Field-specific reporting

Please select the one below that is the best fit for your research. If you are not sure, read the appropriate sections before making your selection.

☒ Life sciences ☐ Behavioural & social sciences ☐ Ecological, evolutionary & environmental sciences

For a reference copy of the document with all sections, see nature.com/documents/nr-reporting-summary-flat.pdf

Life sciences study design

All studies must disclose on these points even when the disclosure is negative.

Sample size	At least 30 nurse cells are counted for the calculation of FRAP results. 30 ovaries are used for total RNA-seq; 100 ovaries are used for the ovaries IP. One 6mm plate S2 cell with 100% confluency is used for the co-IP. One 10mm plate S2 cell with 100% confluency is used for the in vitro methylation.
Data exclusions	No data were excluded.
Replication	For the RT-qPCR, 2 biological and 3 technical replicates are included. Western blot results are repeated twice. In vitro methylation assay are performed twice . Limited protease assay are repeated twice.
Randomization	nurse cells at stage between 6-9 are selected for the FRAP experiments. Flies hatched rate is calculated using random male and female flies; Random young female flies are selected and feed yeast for the RT-qPCR, IP and IP-seq experiments.
Blinding	No blinding was used.

Reporting for specific materials, systems and methods

We require information from authors about some types of materials, experimental systems and methods used in many studies. Here, indicate whether each material, system or method listed is relevant to your study. If you are not sure if a list item applies to your research, read the appropriate section before selecting a response.

Materials & experimental systems

n/a	Involved in the study	
☐	☒	Antibodies
☐	☒	Eukaryotic cell lines
☒	☐	Palaeontology and archaeology
☐	☒	Animals and other organisms
☒	☐	Human research participants
☒	☐	Clinical data
☒	☐	Dual use research of concern

Methods

n/a	Involved in the study	
☒	☐	ChIP-seq
☒	☐	Flow cytometry
☒	☐	MRI-based neuroimaging

Antibodies

Antibodies used	mouse anti-GFP (Santa Cruz) sc-390394;rabbit SYM11 antibody (Sigma Aldrich)07-413; mouse anti-FLAG M2 (Sigma Aldrich)F1804; mouse anti-HA (Sigma Aldrich) H9658;
Validation	Antibodies used in this study are commercially available,the specificity had been tested by the supplier.

Eukaryotic cell lines

Policy information about cell lines

Cell line source(s)	S2R+ flybase ID FBtc0000150
Authentication	not authenticated
Mycoplasma contamination	cell lines tested are negative for mycoplasma contamination
Commonly misidentified lines (See ICLAC register)	no misidentified lines are used

Animals and other organisms

Policy information about studies involving animals; ARRIVE guidelines recommended for reporting animal research

Laboratory animals	fruit fly (Drosophila melanogaster)
Wild animals	no wild animals
Field-collected samples	no field-collected samples
Ethics oversight	Project is approved by the California institute of technology

Note that full information on the approval of the study protocol must also be provided in the manuscript.
