# Supplementary material for: Binding of guide piRNA triggers methylation of the unstructured N-terminal region of Aub leading to assembly of the piRNA amplification complex
Source: Nat Commun. 2021 Jul 1;12:4061. doi: 10.1038/s41467-021-24351-x (PMC8249470; doi:10.1038/s41467-021-24351-x)
Supplement: Supplementary file 2 — Peer Review File [file 41467_2021_24351_MOESM2_ESM.pdf]

REVIEWER COMMENTS

Reviewer #1 (Remarks to the Author):

In this manuscript, Huang X et al reported that Krimper uses its N-terminal eTudor domain (eTud1) to bind Ago3 in a methylation- independent manner and methylation of the target arginine residues will disrupt binding, and use the eTud2 domain to recognize the methylated N-terminus of Aub and this binding depends on sDMA of the target arginine resideus. They further show that sDMA modification of Aub is required for piRNA biogenesis through the ping-pong cycle but is dispensable for loading of piRNA into Aub. RNA binding promotes Aub arginine methylation, and triggers conformational change in Aub, exposing its N terminus to the methyltransferase Csul/Vls complex. This is a very interesting piece of work, which represents some exciting advance in the field. Overall, this paper is well written, and the data are of solid quality. However, I have some concerns, which should be addressed prior to publication.

1.What is the structural explanation why eTud1 cannot bind to unmethylated Aub-1 peptide now that it also contains a similar GRGRAR motif found in Ago3-2? By the way, in Fig. 1e, the Aub peptide is labelled as Aub-1, but as aub in Fig. 1f.
2.Why does eTud1 not bind Ago3-1 at all? Does it mean that more than 1 arginine residue in the motif is required in binding?
3.eTub2 binds tighter to methylated Ago3-2 than to methylated Aub, is there any biological significance, such as methylated Ago3-2 can compete out Aub to release Aub
4. A sequence alignment should be made with the TDRD2 eTudor to show if there is any sequence conservation, especially for the residues involved in binding the first 2 arginine residues in the TDRD2-PIWIL1 structure, which are the major structural determinants in the binding.
5. Although the manuscript is well written, but there are still many grammar errors, which need to be fixed as well.

Jinrong Min

Reviewer #2 (Remarks to the Author):

The symmetric dimethylarginine (sDMA) modification is widely present in unstructured N-terminal region of Piwi proteins, which is known critical for Piwi binding to Tudor domain-containing proteins. Nevertheless, the regulatory function of such modification in piRNA pathway has not been fully understood. In this manuscript, Huang et al. showed that piRNA binding triggers sDMA of Aub, while such Aub-sDMA is essential for piRNA biogenesis and transposon silencing. First, they showed that Krimper interacts simultaneously with Aub and Ago3 by two separate Tudor domains, with specificity for methylation state of Aub. By structural studies, they revealed the two Tudor domains of Krimper showing distinct architectures that are responsible for differential binding of sDMA modified Aub and unmodified Ago3. Next, the in vivo assays showed that sDMA of Aub is required for ping-pong cycle, transposon silencing and fertility of Drosophila. Finally, they found that piRNA binding triggers protein conformational change in Aub, exposing its N terminus to the PRMT5 methylosome complex. Overall, most of the experiments are well done, and many of the data presented are convincing and support the conclusions. Following comments may help for further improvement.

Major points
1. I suggest to test if the methylation-deficient mutant Aub (mdAub) exhibits a normal slicer activity, which could exclude the potential effect of cleavage defect on the Ping-Pang cycle.
2. It is surprised to see that Aub was still methylated in Fig 4g lane 2 and interacted with the

methylosome complex in Fig 5 in piRNA-free S2 cells, given that piRNA-loading is essential for sDMA of Aub.
3. I am wondering if loading of piRNA precursor could also induce the conformational change and sDMA methylation of Aub. The PAZmut mutant, which is deficient in mature piRNA loading but could bind precursors (EMBO Reports. 2020), could be a good one to test this.
4. In Fig 3a and 4a, the author should include the IB panels to show whether the mutations alter Aub expression in flies.
Minor points
1. Overall, the labelling in western blot panels should be improved.
2. In Fig 5d, the labeling of lanes 3 and 4 should be be "md" rather than "rd"?

Reviewer #3 (Remarks to the Author):

In this manuscript, Huang and Hu et al. show that the two Tudor domains of Krimper (Tud1 and Tud2) bind unmodified Ago3 and sDMA-modified Aub, respectively, and that the sDMA modification of Aub is regulated by loading of a piRNA guide, thereby dynamically assembling the piRNA amplification complex. In general, the experiments are well executed and the data support the conclusion. The following points should be addressed before publication.

Major points:
1. The small RNA seq data in Fig. 3f should be more thoroughly analyzed. 1) According to the result in Fig 3g, the authors should not simply focus on the top 20 most abundant TE families but should appropriately categorize the piRNA clusters in their analysis. 2) Rather than just focusing on sense & 10A & non-1U piRNAs, the data for antisense piRNAs should also be presented and the 1U/10A bias should be more rigorously analyzed. 3) The ping-pong pair factions of some transposons seem to be well rescued by mdAub. How do the authors explain this observation? What is the common feature(s) among them (e.g., more dependent on homotypic ping-pong)?

Minor points:
1. The authors have previously shown that Aub and Ago3 interact with both Tud1 and Tud2 (2015 Mol Cell, 59, 564-575). The apparent discrepancy should be more carefully explained. Specifically, does the Aub peptide fragment containing R26 bind to Tud1?
2. The authors should specify the tools used in their bioinformatic analyses and also how small RNA seq data were normalized.
3. In Fig 3i upper panel, the wtAub data should be presented on the right of the mdAub data.
4. In Fig 4g, h, sDMA modification is detected in the absence of 26-nt ssRNA. Does this mean that FLAG-Aub is partially loaded with endogenous RNA fragments in S2 cells?
5. In Fig. 5d: "rd" should read "md".
6. Exd Data Fig. 5a requires molecular markers.
7. The order of Figs and Exd Data Figs should be amended so that they are sequentially cited in the text.
8. The authors should discriminate PIWI (subfamily name) and Piwi (Drosophila protein name) throughout the text.
9. Page 3, line 7: "Argonautes bind nucleic acids guides" should read "Argonautes bind nucleic acid guides".
10. Page 3, line 6 from the bottom: "produces symmetrically methylated arginine (sDMA) residues" should read "produces symmetrically dimethylated arginine (sDMA) residues".
11. Page 5, line 1: "Krimper that lacks the N-terminal self-interaction region (residues 1-301aa)" should probably read "residues 1-300 aa" (in Fig. 1c, ΔN-Krimp is shown as 301-746).
12. Page 10, line 2 from the bottom: the underlines in "piRNA binding" make the abbreviation "pbAub", not "pdAub".

13. The discussion part is reasonable but rather lengthy.

We are very grateful to all three reviewers for their valuable criticism and suggestions, which we believe have greatly contributed to improving the manuscript. We performed new experiments and analyses as well as implemented changes in the text of the paper to address the comments. Below, we provide our point-by-point response (shown in blue) to the comments of each reviewer.

Reviewer #1

In this manuscript, Huang X et al reported that Krimper uses its N-terminal eTudor domain (eTud1) to bind Ago3 in a methylation- independent manner and methylation of the target arginine residues will disrupt binding, and use the eTud2 domain to recognize the methylated N-terminus of Aub and this binding depends on sDMA of the target arginine resideus. They further show that sDMA modification of Aub is required for piRNA biogenesis through the ping-pong cycle but is dispensable for loading of piRNA into Aub. RNA binding promotes Aub arginine methylation, and triggers conformational change in Aub, exposing its N terminus to the methyltransferase Csul/Vls complex. This is a very interesting piece of work, which represents some exciting advance in the field. Overall, this paper is well written, and the data are of solid quality. However, I have some concerns, which should be addressed prior to publication.

1.What is the structural explanation why eTud1 cannot bind to unmethylated Aub-1 peptide now that it also contains a similar GRGRAR motif found in Ago3-2? By the way, in Fig. 1e, the Aub peptide is labelled as Aub-1, but as aub in Fig. 1f.

Although the overall topology of eTud1 is similar to the other extended Tudor domains, eTud1 has its own unique folding. It forms both a hydrophilic and a hydrophobic concave region in the cleft between the Tudor and SN-like subdomains of eTud1 (Fig. 2h). The AGO3-2 peptide forms an a-helical

structure which fits into the narrow binding cleft of eTud1. The N-terminal AUB (NH2-NPVIARGRGRGRK-COOH), sequence with an additional “GR” motif appears to be more hydrophilic than AGO3-2 and may not readily fit into the narrow hydrophobic concave cleft of eTud1. We have added this comparison in the text of the revised manuscript.
We thanked the reviewer for pointing out the typo and we corrected it as suggested (Fig. 1f).

2.Why does eTud1 not bind Ago3-1 at all? Does it mean that more than 1 arginine residue in the motif is required in binding?

As stated in the Q1, unlike Ago3-2, Ago3-1 with only one “GR” motif appears to be unable to form strong interactions with eTud1. We have added this comparison in the main text.

3.eTub2 binds tighter to methylated Ago3-2 than to methylated Aub, is there any biological significance, such as methylated Ago3-2 can compete out Aub to release Aub

According to previous findings by others (DOI:https://doi.org/10.1016/j.molcel.2015.06.024) and us (DOI:https://doi.org/10.1016/j.molcel.2015.07.017), Krimper only interacts with unmethylated Ago3 *in vivo*. Thus, it seems that *in vivo* other factors prevent methylated Ago3 from interacting with Krimp.

4. A sequence alignment should be made with the TDRD2 eTudor to show if there is any sequence conservation, especially for the residues involved in binding the first 2 arginine residues in the TDRD2-PIWIL1 structure, which are the major structural determinants in the binding.

We have made sequence alignment between TDRD2 and eTud1 of Krimper and show it on the new Extended Data Fig. 3b. Although there is some sequence conservation between TDRD2 and Krimper eTud1, residues involved in peptide recognition are not well conserved. Only two peptide-recognizing residues are conserved between TDRD2 and Krimper eTud1. Phe391 and Asp393 in TDRD2, which participate in recognizing PIWIL1 Arg10, are conserved with Krimper eTud1 Phe400 and Glu402, which recognize AGO3-2 Arg70.

5. Although the manuscript is well written, but there are still many grammar errors, which need to be fixed as well.

We thanke the reviewer for pointing out grammar mistakes and we corrected them as suggested.

Jinrong Min

Reviewer #2 (Remarks to the Author):

The symmetric dimethylarginine (sDMA) modification is widely present in unstructured N-terminal region of Piwi proteins, which is known critical for Piwi binding to Tudor domain-containing proteins. Nevertheless, the regulatory function of such modification in piRNA pathway has not been fully understood. In this manuscript, Huang et al. showed that piRNA binding triggers sDMA of Aub, while such Aub-sDMA is essential for piRNA biogenesis and transposon silencing. First, they showed that Krimper interacts simultaneously with Aub and Ago3 by two separate Tudor domains, with specificity for methylation state of Aub. By structural studies, they revealed the two Tudor domains of Krimper showing distinct architectures that are responsible for differential binding of sDMA modified Aub and unmodified Ago3. Next, the in vivo assays showed that sDMA of Aub is required for ping-pong cycle, transposon silencing and

fertility of Drosophila. Finally, they found that piRNA binding triggers protein conformational change in Aub, exposing its N terminus to the PRMT5 methylosome complex. Overall, most of the experiments are well done, and many of the data presented are convincing and support the conclusions. Following comments may help for further improvement.

Major points

1. I suggest to test if the methylation-deficient mutant Aub (mdAub) exhibits a normal slicer activity, which could exclude the potential effect of cleavage defect on the Ping-Pang cycle.

We thank the reviewer for this suggestion. As suggested, we have performed *in vitro* assays to compare slicer activities of the wild-type and mdAub proteins. The results show that mdAub exhibits a normal slicer activity similar to wild-type Aub. We added this result to revised manuscript (Extended Data Fig. 4d)

2. It is surprised to see that Aub was still methylated in Fig 4g lane 2 and interacted with the methylosome complex in Fig 5 in piRNA-free S2 cells, given that piRNA-loading is essential for sDMA of Aub.

There are two non-mutually exclusive explanantions for these results: (a) a fraction of Aub molecules expressed in S2 cells might be loaded with cellular RNA (b) a fraction of Aub molecules might be modified by the methylosome complex even if they lack RNA guides due to random expose of their N-termini to the methylosome complex. Our results (Extended Data Fig. 4d) and experiments from other groups demonstrate that Aub can bind any 5'phosphorylated RNA of proper length and use it as a guide *in vitro* or in cell extract. Therefore, it is plausible that Aub finds opportunities to bind cellular RNA present in S2 cells, such as tRNA fragments known to be bound by PIWI proteins in various systems. To explore if Aub is loaded with guide RNA in S2

cells, we labeled nucleic acids associated with purified protein and found that a small amount of RNAs of various sizes is indeed bound by Aub (data not shown). Thus, Aub binding of RNA in S2 cells might promote its methylation even in the absence of genuine piRNA. This conclusion is further supported by the observation that sDMA modification is suppressed in the Aub mutant deficient in RNA binding (pdAub) if expressed in S2 cells (Fig. 6b). However, it should be noted that pdAub expressed in germ or S2 cells still has miniscule but detectable levels of sDMA modification (Fig. 5a, 6b, compare pdAub with mdAub), indicating that the correlation between piRNA binding and methylation is not absolute. We also observed that insertion of a heterologous sequence between the N-terminus and the rest of Aub renders it a good substarte for sDMA modification even if it does not bind RNA (Fig 6a). Furthermore, we generated new Aub PAZ mutant lacking the 3'-end piRNA binding residues and found that it failed to be modified, further confirming the role of guide binding in promoting sDMA modification (Fig. 5i). Overall, the results from several experimental systems together indicate that RNA loading strongly promotes sDMA modification, however, while binding of genuine piRNAs induce modification in germ cells, other RNAs can substitute for genuine piRNAs leading to modification in heterologous systems.

3. I am wondering if loading of piRNA precursor could also induce the conformational change and sDMA methylation of Aub. The PAZmut mutant, which is deficient in mature piRNA loading but could bind precursors (EMBO Reports. 2020), could be a good one to test this.

We are thankful for suggesting this experiment that allowed us to further test requirements for sDMA modification. We expressed Aub PAZmut mutant, which is deficient in 3' end binding but could bind 5' end of precursors and tested its methylation level upon loading with synthetic 26nt ssRNA. We found that unlike the wild-type protein, Aub PAZmut has miniscule level of sDMA

modification (Fig 5i), indicating that complete binding of the guide RNA – both its 5’ and 3’end – is required to induce modification. We added this result to the Results and Discussion sections of the revised manuscript.

4. In Fig 3a and 4a, the author should include the IB panels to show whether the mutations alter Aub expression in flies.

We have added panels to show the amount of wild-type, mdAub and pdAub Aub in ovarian lysates to the new Extended Data Fig. 4a. mdAub is expressed at a level similar to the wild-type protein, while expression of pdAub, which lacks piRNA-binding capacity is diminished.

Minor points

1. Overall, the labelling in western blot panels should be improved.

We have re-labelled western blot panels to clarify source of material, antibodies used for IP and Western.

2. In Fig 5d, the labeling of lanes 3 and 4 should be be “md” rather than “rd”?

Thank you for noticing, we have corrected this mistake.

<u>Reviewer #3 (Remarks to the Author):</u>

In this manuscript, Huang and Hu et al. show that the two Tudor domains of Krimper (Tud1 and Tud2) bind unmodified Ago3 and sDMA-modified Aub, respectively, and that the sDMA modification of Aub is regulated by loading of a piRNA guide, thereby dynamically assembling the piRNA amplification complex. In general, the experiments are well executed and the data support the conclusion. The following points should be addressed before publication.

Major points:

1. The small RNA seq data in Fig. 3f should be more thoroughly analyzed. 1) According to the result in Fig 3g, the authors should not simply focus on the top 20 most abundant TE families but should appropriately categorize the piRNA clusters in their analysis. 2) Rather than just focusing on sense & 10A & non-1U piRNAs, the data for antisense piRNAs should also be presented and the 1U/10A bias should be more rigorously analyzed. 3) The ping-pong pair factions of some transposons seem to be well rescued by mdAub. How do the authors explain this observation? What is the common feature(s) among them (e.g., more dependent on homotypic ping-pong)?

We are thankful for suggesting additional analyses, which we have included in the revised manuscript.

1) We have performed comprehensive analysis of piRNA clusters throughgout the whole genome. The analysis showed that piRNAs from all dual-strand clusters expressed in the germline are greatly reduced in flies expressing mdAub. These data are shown in new figure 4c and described in the text.

2) We have performed additional analyses of piRNAs and incorporated these results in the revised manuscript. We show both sense and antisense piRNA in revised Fig. 4b. We also analyzed the 1U and 10A biases in piRNAs mapping to individual TE families (shown in revised Fig. 4b) and for all repeat-derived piRNAs (shown in new Fig. 4e and Extended Data Fig. 5a). Additional analyses further support the conclusion that expression of mdAub fails to rescue ping-pong piRNA processing.

3) While performing piRNA analysis for the revision, we have found a mistake in processing of data for Fig. 3f (Fig. 4b in the revised manuscript), which shows fractions of ping-pong pairs for different TE families. The corrected figure provided in the revised manuscript indicates that fractions of ping-pong piRNA pairs are decreased in mdAub relative to control for all analyzed TE families. For the majority of TE families, expression of wtAub rescues the decrease in

the number of ping-pong pairs seen in aub mutants, while expression of mdAub provides only partial rescue. One TE family, element 297, shows anomalous behavior with similar decease of ping-pong pairs in both wtAub and mdAub. Notably, the rescue by mdAub and wtAub also has a similar effect on the total abundance of piRNAs mapping to the element 297. At this point we are unable to explain the anomalous behavior of element 297.

Minor points:

1. The authors have previously shown that Aub and Ago3 interact with both Tud1 and Tud2 (2015 Mol Cell, 59, 564-575). The apparent discrepancy should be more carefully explained. Specifically, does the Aub peptide fragment containing R26 bind to Tud1?

Indeed, previously we have shown that Aub can bind three different Krimp fragments: N-domain+Tud1, Tud1+Tud2 and Tud2. However, the data presented in this manuscript indicate that Aub interacts with Tud2 exclusively. To investigate this discrepancy we have repeated co-IP experiments described in the previous paper and also tested additional Krimp fragments. Our results confirmed the previous observation that both N+Tud1 and Tud2 fragments coimmunoprecipitate with Aub, however, we also found that the Tud1 fragment alone (without the N-terminal domain) does not co-IP with Aub. These results explain the discrepancy as they demonstrate that Tud1 does not bind Aub and the presence of the N-terminal domain of Krimp used in the previous paper is responsible for interaction. Krimp's N-terminal domain is responsible for self-interaction and we also found that Krimp protein is expressed in S2 cells. Thus, co-immunoprecipitation of Aub and the Krimp N+Tud1 fragment can be explained by formation of a tertiary complex that contains full-length Krimp protein present in S2 cells. In this complex Aub binds the Tud2 domain of full-length Krimp, which binds the N+Tud1 fragment. We incorporated this result in the revised manuscript (Extended Data Fig. 1b).

On the other hand, there is no discrepancy between our new findings and previous results regarding interaction between Ago3 and Krimp. Previously, we showed that in S2 cells Ago3 co-IPs with both N+Tud1 and Tud2 fragments (though strongest interaction was observed for the fragement containing both Tud1+Tud2 domains). The new findings also demonstrate that sDMA-modified, but not unmodified Ago3 peptides bind Tud2 *in vitro* (Extended Data Fig.1a). However, as was shown previously, in germ cells Krimp complexes contain unmodified Ago3, which according to our data can only bind to Tud1 domain. We discuss these results and possible reasons for the difference between the *in vitro* and *in vivo* results in the manuscript.

2. The authors should specify the tools used in their bioinformatic analyses and also how small RNA seq data were normalized.

We have added the information about bioinformatic tools and RNA-seq data normalization in the Material and Method section.

3. In Fig 3i upper panel, the wtAub data should be presented on the right of the mdAub data.

We have fixed the presentation of data on Fig. 3i (new Fig. 4g) as suggested.

4. In Fig 4g, h, sDMA modification is detected in the absence of 26-nt ssRNA. Does this mean that FLAG-Aub is partially loaded with endogenous RNA fragments in S2 cells?

The similar question was also raised by reviewer #2 (point 2), so we provide a combined answer:

There are two non-mutually exclusive explanantions for these results: (a) a fraction of Aub molecules expressed in S2 cells might be loaded with cellular RNA (b) a fraction of Aub molecules might be modified by the methylosome complex even if they lack RNA guides due to random expose of their N-termini to the methylosome complex. Our results (Extended Data Fig. 4d) and experiments from other groups demonstrate that Aub can bind any 5'phosphorylated RNA of proper length and use it as a guide *in vitro* or in cell extract. Therefore, it is plausible that Aub finds opportunities to bind cellular RNA present in S2 cells, such as tRNA fragments known to be bound by PIWI proteins in various systems. To explore if Aub is loaded with guide RNA in S2 cells, we labeled nucleic acids associated with purified protein and found that a small amount of RNAs of various sizes is indeed bound by Aub (data not shown). Thus, Aub binding of RNA in S2 cells might promote its methylation even in the absence of genuine piRNA. This conclusion is further supported by the observation that sDMA modification is suppressed in the Aub mutant deficient in RNA binding (pdAub) if expressed in S2 cells (Fig. 6b). However, it should be noted that pdAub expressed in germ or S2 cells still has miniscule but detectable levels of sDMA modification (Fig. 5a, 6b, compare pdAub with mdAub), indicating that the correlation between piRNA binding and methylation is not absolute. We also observed that insertion of a heterologous sequence between the N-terminus and the rest of Aub renders it a good substarte for sDMA modification even if it does not bind RNA (Fig 6a). Furthermore, we generated new Aub PAZ mutant lacking the 3'-end piRNA binding residues and found that it failed to be modified, further confirming the role of guide binding in promoting sDMA modification (Fig. 5i). Overall, the results from several experimental systems together indicate that RNA loading strongly promotes sDMA modification, however, while binding of genuine piRNAs induce modification in germ cells, other RNAs can substitute for genuine piRNAs leading to modification in heterologous systems.

5. In Fig. 5d: “rd” should read “md”.

Thank you, we have corrected the error.

6. Exd Data Fig. 5a requires molecular markers.

We have modified the figure to show molecular markers (updated Extended Fig. 6a).

7. The order of Figs and Exd Data Figs should be amended so that they are sequentially cited in the text.

We have corrected the figures to align panel order with the text.

8. The authors should discriminate PIWI (subfamily name) and Piwi (Drosophila protein name) throughout the text.

We have chaged the text throughout as suggested to discriminate subfamily and protein names.

9. Page 3, line 7: “Argonautes bind nucleic acids guides” should read “Argonautes bind nucleic acid guides”.

Thank you for noticing, we have corrected the text as suggested.

10. Page 3, line 6 from the bottom: “produces symmetrically methylated arginine (sDMA) residues” should read “produces symmetrically dimethylated arginine (sDMA) residues”.

Thank you for noticing, we have corrected the text as suggested.

11. Page 5, line 1: “Krimper that lacks the N-terminal self-interaction region (residues 1-301aa)” should probably read “residues 1-300 aa” (in Fig. 1c, ΔN-Krimp is shown as 301-746).

Thank you for noticing, we have corrected the text as suggested.

12. Page 10, line 2 from the bottom: the underlines in “piRNA binding” make the abbreviation “pbAub”, not “pdAub”.

Thank you for pointing this out, we have corrected it as suggested.

13. The discussion part is reasonable but rather lengthy.

We have shorted and streamlined the Discussion.

REVIEWERS' COMMENTS

Reviewer #1 (Remarks to the Author):

The authors have addressed all of my concerns in the revision, so I support its publication.

Reviewer #2 (Remarks to the Author):

Most of concerns have been adequately addressed by additional data in revised version of manuscript.
Minor point:
The labelling in western blot panels, including molecular markers, should be improved.

Reviewer #3 (Remarks to the Author):

The authors have adequately addressed most of my previous concerns. The following minor points should be fixed before publication.

1. In the rebuttal letter, the authors state "1) We have performed comprehensive analysis of piRNA clusters throughgout the whole genome. The analysis showed that piRNAs from all dual-strand clusters expressed in the germline are greatly reduced in flies expressing mdAub. These data are shown in new figure 4c and described in the text." However, there are three problems. 1) The main text does not contain any reference to Fig 4c. I assume that "Unlike the wild-type protein, expression of mdAub fails to rescue piRNA generation throughout the genome indicating that sDMA is crucial for piRNA biogenesis (Fig 4b and 4d)." in page 10 is the corresponding part. 2) The legend for Fig 4c says "Genomic windows with more than 5 RPM in libraries from heterozygous ovaries and more than 80% reduction in aub mutant were selected for further analysis." If this statement is correct, the authors have specifically selected genomic windows with >80% reduction of piRNAs, and then claimed that piRNAs from those regions are greatly reduced (which is too natural to be claimed). Isn't it much more important that piRNA production from those regions cannot be rescued by mdAub? 3) In Fig. 4d, 42AB and 38C show log2FC=~0 for the wtAub rescue, but those regions are quite green in Fig. 4c. Why?
2. Page 10, In ovaries of control (aub heterozygotes) flies piRNA are twice as abundant as miRNA: "piRNAs" and "miRNAs" should be plural.
3. Page 13, However, loading and final processing of piRNA guides is coupled, so that longer piRNA precursors are first anchored at their 5' end, followed by trimming of their 3' end before they can be anchored by the PAZ domain: Please note that, in flies, 3' trimming does not always happen.
4. Page 32, Protein level were detected by WB: "Levels" should be plural (or "the protein level was detected...").

We are very grateful to all three reviewers for their valuable criticism and suggestions, which we believe have greatly contributed to improving the manuscript. Below, we provide our point-by-point response (shown in blue) to the comments of each reviewer.

Reviewer #2 (Remarks to the Author):

Most of concerns have been adequately addressed by additional data in revised version of manuscript.
Minor point:
The labelling in western blot panels, including molecular markers, should be improved.
Molecular weight for all western blots were added as suggested.

Reviewer #3 (Remarks to the Author):

The authors have adequately addressed most of my previous concerns. The following minor points should be fixed before publication.

1. In the rebuttal letter, the authors state “1) We have performed comprehensive analysis of piRNA clusters throughout the whole genome. The analysis showed that piRNAs from all dual-strand clusters expressed in the germline are greatly reduced in flies expressing mdAub. These data are shown in new figure 4c and described in the text.” However, there are three problems.

1) The main text does not contain any reference to Fig 4c. I assume that “Unlike the wild-type protein, expression of mdAub fails to rescue piRNA generation throughout the genome indicating that sDMA is crucial for piRNA biogenesis (Fig 4b and 4d).” in page 10 is the corresponding part.

We apologized for this mistake. Figure references were added in the text. “Almost all regions that generate piRNAs with notable exception of uni-strand piRNA clusters such as *flam* and *20A* show dramatic loss of piRNA in the $aub^{HN/QC}$ mutant (Fig. 4c). Unlike the wild-type protein, expression of mdAub fails to rescue piRNA generation throughout the genome indicating that sDMA is crucial for piRNA biogenesis (Fig. 4b, 4c and 4d).”

2) The legend for Fig 4c says “Genomic windows with more than 5 RPM in libraries from heterozygous ovaries and more than 80% reduction in aub mutant were selected for further analysis.” If this statement is correct, the authors have specifically selected genomic windows with >80% reduction of piRNAs, and then claimed that piRNAs from those regions are greatly reduced (which is too natural to be claimed). Isn’t it much more important that piRNA production from those regions cannot be rescued by mdAub?

We found that mdAub couldn’t rescue piRNA derived from genomic region affected by aub mutant using genome wide analysis. We modified our figure legends for Fig. 4c to clarify the result as below “ Aub sDMA modification is required for generation of piRNAs from piRNA clusters. Reads from 5 kb genomic windows were normalized to total miRNAs reads count. Heatmap shows piRNA fold change compared to control (*aub* heterozygotes). wtAub, but not mdAub rescues piRNA expression from genomic regions affected by *aub* mutation. Genomic windows with more than 5 RPM in control and more than 80% reduction in aub mutant are shown; uni-strand clusters flam and 20A that are not affected in *aub* mutants are shown for comparison.”

3) In Fig. 4d, 42AB and 38C show log2FC=~0 for the wtAub rescue, but those regions are quite green in Fig. 4c. Why?

Thanks for pointing out the discrepancy.

There are two reasons.

1. Overall, there are slightly more piRNAs compared to miRNAs in wtAub rescue compared to control (~1.5 fold in Fig. 4a) that lead to slightly elevated levels of piRNA from all regions on Fig. 4c. The same trend is presented on revised Fig. 4d: piRNAs in wtAub rescue are generally have log2(wtAub/control) values slightly above 0. The total normalized uniquely genome mapped piRNAs reads count in wtAub rescue is around 1.5-fold compared to control.

2. The color bar in the Fig. 4c was mislabeled. Should be -5(Red) to 5(Green). And the bars indicating region of cluster (42AB, 38C, 80EF) weren’t correctly labeled. We revised them in the new Fig. 4c.

2. Page 10, In ovaries of control (aub heterozygotes) flies piRNA are twice as abundant as miRNA: “piRNAs” and “miRNAs” should be plural.

Thanks for pointing out mistake. We changed it as suggested.

3. Page 13, However, loading and final processing of piRNA guides is coupled, so that longer piRNA precursors are first anchored at their 5’ end, followed by trimming of their 3’ end before they can be anchored by the PAZ domain: Please note that, in flies, 3’ trimming does not always happen.

Thanks for pointing it out. 3’ end of piRNA can be directly defined by Zuc in fly, in this case, no more trimming is required. Therefore, we modified our sentence in below. “However, loading and final processing of piRNA guides is coupled, so that longer piRNA precursors are first anchored at their 5’ end, followed by Zuc mediated cleavage or trimming of their 3’ end before they can be anchored by the PAZ domain [56,57,60].”

4. Page 32, Protein level were detected by WB: “Levels” should be plural (or “the protein level was detected...”).

Thanks for pointing out the mistake. This sentence was deleted based on editorial request to shorten the figure legend.
